# Profiling PRMT methylome reveals roles of hnRNPA1 arginine methylation in RNA splicing and cell growth

**DOI:** 10.1038/s41467-021-21963-1

**Published:** 2021-03-29

**Authors:** Wen-juan Li, Yao-hui He, Jing-jing Yang, Guo-sheng Hu, Yi-an Lin, Ting Ran, Bing-ling Peng, Bing-lan Xie, Ming-feng Huang, Xiang Gao, Hai-hua Huang, Helen He Zhu, Feng Ye, Wen Liu

**Affiliations:** 1 State Key Laboratory of Cellular Stress Biology, School of Pharmaceutical Sciences, Xiamen University, Xiamen Fujian, China; 2 Fujian Provincial Key Laboratory of Innovative Drug Target Research, School of Pharmaceutical Sciences, Xiamen University, Xiamen Fujian, China; 3 State Key Laboratory of Molecular Vaccinology and Molecular Diagnostics, National Institute of Diagnostics and Vaccine Development in Infectious Diseases, School of Pharmaceutical Sciences, Xiamen University, Xiamen Fujian, China; 4 Department of Pathology, The Second Affiliated Hospital, Shantou University Medical College, Shantou Guangdong, China; 5 Department of Urology, Ren Ji Hospital, School of Medicine and School of Biomedical Engineering, Shanghai Jiao Tong University, Shanghai, China; 6 Department of Medical Oncology, The First Affiliated Hospital of Xiamen University, Fujian, China

**Keywords:** RNA-binding proteins, Proteomics, Methylation, Alternative splicing

## Abstract

Numerous substrates have been identified for Type I and II arginine methyltransferases (PRMTs). However, the full substrate spectrum of the only type III PRMT, PRMT7, and its connection to type I and II PRMT substrates remains unknown. Here, we use mass spectrometry to reveal features of PRMT7-regulated methylation. We find that PRMT7 predominantly methylates a glycine and arginine motif; multiple PRMT7-regulated arginine methylation sites are close to phosphorylations sites; methylation sites and proximal sequences are vulnerable to cancer mutations; and methylation is enriched in proteins associated with spliceosome and RNA-related pathways. We show that PRMT4/5/7-mediated arginine methylation regulates hnRNPA1 binding to RNA and several alternative splicing events. In breast, colorectal and prostate cancer cells, PRMT4/5/7 are upregulated and associated with high levels of hnRNPA1 arginine methylation and aberrant alternative splicing. Pharmacological inhibition of PRMT4/5/7 suppresses cancer cell growth and their co-inhibition shows synergistic effects, suggesting them as targets for cancer therapy.

## Abstract (web summary)

Arginine methyltransferases (PRMTs) are involved in the regulation of various physiological and pathological conditions. Using proteomics, the authors here profile the methylation substrates of PRMTs 4, 5 and 7 and characterize the roles of these enzymes in cancer-associated splicing regulation.

## Introduction

The epigenome regulates gene expression, which has been shown to be critical for cell growth, differentiation, and development and disease progression^1^. Epigenetic changes could arise from a variety of modifications on DNA, RNA, and histones. For instance, histones can be modified by acetylation, methylation, phosphorylation, and ubiquitination, among others^2^. Arginine methylation was first discovered in the early 1970s^3,4^ and was later recognized as a widespread post-translational modification (PTM) in many proteins^5–8^. It plays important roles in signaling transduction, DNA damage repair, gene transcription, splicing regulation, and RNA metabolism, among others^9^. In mammals, arginine methylation is catalyzed by the family of arginine methyltransferases (PRMT), which has at least nine members, i.e., PRMT1 to 9^10^. PRMTs can modify the guanidinium group of arginine residues, producing ω-N^G^-mono-methyl-arginine (MMA), ω-N^G^, N^G^-asymmetric di-methyl-arginine (aDMA), or ω-N^G^, N^G^-symmetric di-methyl-arginine (sDMA)^2^. PRMTs are classified into three types according to the final methyl-arginine products generated: type I PRMTs include PRMT1, 2, 3, 4, 6, and 8 that catalyze the formation of MMA and aDMA; type II PRMTs include PRMT5 and PRMT9 that catalyze the formation of MMA and sDMA; and type III PRMT include PRMT7 that catalyzes the formation of MMA^10^.

Owing to the functional importance of arginine methylation in many proteins, PRMTs have been reported to be involved in the transcriptional and post-transcriptional regulation of gene expression, mRNA processing and translation, and intracellular signaling during development and disease progression, particularly in cancers^10,11^. For instance, the PRMT1-mediated asymmetric di-methylation of histone H4 arginine 3 (H4R3me2a), as well as a number of non-histone proteins, such as C/EBPα^12^, Twist1^13^ and Gli1^14^, promote transcription, whereas the PRMT5-mediated symmetric di-methylation of proteins is associated with the inactivation of transcription^15–17^. The functional role of PRMTs in RNA splicing regulation has been highlighted by a large number of splicing factors, such as SF3B2^18^, hnRNPA1^19^, SRSF2^8^, SAP145^20^, SmD1, and SmD3^21,22^ that are regulated by arginine methylation. The aberrant expression and/or activity of PRMTs has been observed in a variety of human diseases, particularly in cancers^5,9,11,23^. Therefore, the PRMT family has emerged as a class of therapeutic targets in drug discovery and cancer treatment^24–26^.

The function of PRMTs in both physiological and pathological conditions is often dependent on their methyltransferase activity. Therefore, unveiling the full scope of their substrates is key to understanding their functions and underlying molecular mechanisms. Among the members of the PRMT family, PRMT1 and PRMT5 have been suggested to contribute to the formation of most of aDMA and sDMA in human cells, respectively^27,28^. The global identification of the substrates of PRMT1, PRMT4, and PRMT5 has been reported in several studies, thus greatly increasing our understanding of arginine methylation and its cellular functions^6–8^. Surprisingly, the number of putative PRMT1, PRMT4, and PRMT5 substrates was very similar^6–8,29^. Recently, Hanghandish et al. have performed quantitative mass spectrometry experiments to identify PRMT7-interacting proteins, which led to the discovery of the PRMT7 direct interaction with eIF2α and its methylation^30^. More recently, Szewczyk et al. reported the identification of 24 unique proteins whose monomethylation are responsive to PRMT7 knockout in HCT116 cells^31^. However, the full scope of arginine methylation catalyzed by PRMT family in the same cellular context remains unknown, hindering our understanding of the arginine methylation network and function.

In this study, employing high-resolution mass spectrometry, we systematically profile arginine methylation regulated by PRMT7 in human embryonic kidney 293 (HEK293) cells. We show that PRMT7 knockdown decreases monomethylation at 297 arginine sites of 174 proteins, which can therefore be considered putative PRMT7 substrates. The PRMT7 methylome is enriched in proteins associated with the spliceosome, RNA transport, mRNA surveillance pathway, and herpes simplex infection. Comparison of the PRMT7, PRMT4, and PRMT5 methylomes shows that they have a large number of unique substrates while sharing a cohort of substrates implicated in mRNA splicing. Consistently, their knockdown causes dramatic changes in alternative splicing in thousands of genes, a few hundreds of which are commonly regulated by all three PRMTs and cancer-relevant. We demonstrate that arginine methylation of the splicing factor hnRNPA1 – a common substrate of PRMT4, 5, and 7—is involved in hnRNPA1 binding to pre-mRNA and the regulation of alternative splicing events commonly regulated by all three PRMTs. PRMT4, 5, and 7 and hnRNPA1 arginine methylation are found to be overexpressed in multiple types of human cancers (including breast, prostate, and colon cancer) and associated with altered cancer-relevant alternative splicing events. Lastly, we show that their pharmacological inhibition effectively suppresses cancer cell growth.

## Results

### Global profiling of PRMT7 substrates in HEK293 cells

**Fig. 1:**
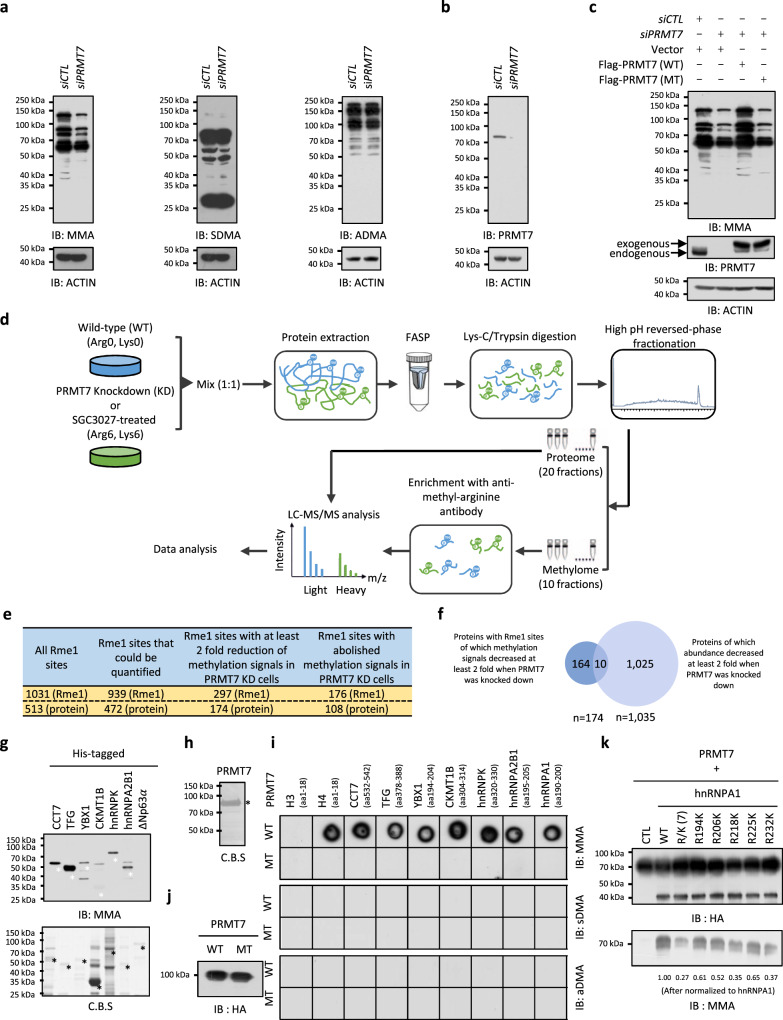
Proteome-wide profiling of arginine methylation regulated by PRMT7. **a**, **b** HEK293 cells transfected with control siRNA (*siCTL*) or siRNA against *PRMT7* (*siPRMT7*) were analyzed by immunoblotting. MMA mono-methyl-arginine, sDMA symmetric di-methyl-arginine, aDMA asymmetric di-methyl-arginine. ACTIN was served as a loading control. **c** HEK293 cells transfected with *siCTL* or *siPRMT7* together with or without Flag-tagged, wild-type (WT) or enzymatic dead mutant (MT) PRMT7 were analyzed by immunoblotting. **d** Experimental flowchart for identification of arginine methylation sites regulated by PRMT7 or responsive to PRMT7 inhibitor SGC3027 in HEK293 cells (see detail in “Methods”). **e** The number of mono-methyl arginine (Rme1) sites detected (column 1), Rme1 sites could be quantified (column 2), Rme1 sites with methylation signals decreased at least two-fold (column 3) or abolished (column 4) when knocking down of PRMT7 was shown. The number of proteins encompass all these methylation sites was also shown (bottom lane). **f** The overlap between PRMT7 methylome and proteins of which abundance was decreased at least two-fold when PRMT7 was knocked down is shown. **g** In vitro methylation assay was performed by mixing purified PRMT7 with CCT7, TFG, YBX1, CKMT1B, hnRNPK, hnRNPA2B1 or △Np63α, followed by immunoblotting with anti-MMA antibody (top panel). Methylation was indicated by white asterisk. The expression of proteins was examined by coomassie blue staining (C.B.S) and indicated by black asterisk (bottom panel). **h** The expression of purified PRMT7 was examined by C.B.S and indicated by black asterisk. **i** In vitro methylation assay was performed by mixing purified PRMT7 with synthetic short peptides from CCT7, TFG, YBX1, CKMT1B, hnRNPK, hnRNPA2B1, and hnRNPA1 proteins. Amino (N)-terminal of histone H3 and H4 were also included. The reactions were subjected to dot blotting. aa, amino acid. **j** The expression of purified PRMT7 was examined by immunoblotting. **k** In vitro methylation assay was performed by mixing purified PRMT7 with hnRNPA1 wild-type (WT) or mutants including R/K (7) (all five arginine (R) residues methylated by PRMT7 were replaced by lysine (K)), R194K, R206K, R218K, R225K, and R232K. The reactions were subjected to immunoblotting. Source data are provided as a Source data file.

We sought out to investigate arginine methylation regulated by PRMT7, the sole type III PRMT, in HEK293 cells. HEK293 cells were transfected with control siRNA and siRNA specific targeting *PRMT7* followed by immunoblotting analysis with anti-mono-methyl-arginine (MMA), asymmetric di-methyl-arginine (aDMA), or symmetric di-methyl-arginine (sDMA)-specific antibodies. These antibodies have been extensively validated and used in other studies^7,8,32–35^. Knockdown of PRMT7 led to a significant decrease in MMA but not in aDMA or sDMA, which was consistent with previous reports showing that the predominant activity of PRMT7 is the mono-methylation of arginine residues in proteins (Fig. 1a)^36–38^. The knockdown efficiency of PRMT7 was examined by immunoblotting (Fig. 1b). The specificity of the siRNA targeting *PRMT7* was demonstrated by rescue experiments, in which wild-type (WT) but not the enzymatic dead mutant (MT)^38,39^ PRMT7 rescued the decrease in MMA levels caused by PRMT7 knockdown (Fig. 1c).

We then employed a high-resolution mass spectrometry (MS) approach to analyze enriched methylated peptides by using anti-MMA antibodies from SILAC (stable isotope labeling by amino acids) labeled wild-type (light) or PRMT7-knockdown (heavy) cells (Fig. 1d). In total, 1031 MMA sites within 513 proteins were predicted, among which 939 MMA sites could be quantified between the control and PRMT7-knockdown cells (Fig. 1e, the first and second column). Upon PRMT7 knockdown, 297 MMA sites from 174 proteins had at least a two-fold reduction in mono-methylation signals, and these 174 proteins were considered as putative substrates (referred to as PRMT7 methylome) (Fig. 1e, the third column, and Supplementary Data 1). In particular, methylation at 176 MMA sites from 108 proteins was completely abolished when PRMT7 was knocked down (Fig. 1e, the fourth column, and Supplementary Data 1). The observed change in the levels of MMA was not a result of the change in the abundance of the corresponding protein as there was nearly no overlap when we compared the putative PRMT7 substrates to PRMT7-regulated proteins deduced from proteomics analysis performed in parallel (Fig. 1f, Supplementary Fig. 1a, and Supplementary Data 2). Indeed, when methylation was normalized to protein levels, only the change in 25 methylation sites (from 20 proteins) was due to the change in protein levels (Supplementary Data 1). In addition, we excluded the possibility that the change in methylation by PRMT7 knockdown was due to the change of other PRMTs, including PRMT1, PRMT3, PRMT4, PRMT5, PRMT6, and PRMT9, as there was no significant change in these PRMTs upon PRMT7 knockdown (Supplementary Data 2). It should be noted that PRMT2 and PRMT8 were undetectable in our proteomics analysis of HEK293 cells.

To further demonstrate that the PRMT7-regulated methylation sites identified by siRNA-mediated knockdown are reliable, we performed methylation profiling in cells treated with or without the PRMT7-specific inhibitor SGC3027^31^. Methylation profiling results revealed that 1126 MMA sites within 524 proteins were confidently predicted, 85.7% of which (*n* = 965) were also detected in the si*PRMT7*-knockdown experiment. This suggests the reproducibility of our MS analysis (*p* < 1e−22) (Supplementary Data 3). Of these 1126 MMA sites, 1027 could be quantified between the control and SGC3027-treated cells (Supplementary Fig. 1b). Upon SGC3027 treatment, 503 MMA sites from 274 proteins had at least two-fold reduction in mono-methylation signals (Supplementary Fig. 1b). Approximately 60% of PRMT7-regulated substrates were also found to be inhibited by SGC3027 (*p* = 2.24e−06), supporting the validity of the putative substrates identified via PRMT7 knockdown.

To further validate the putative PRMT7 substrates identified above, an in vitro methylation assay was performed by mixing purified PRMT7 with randomly selected PRMT7-regulated methylated proteins. All selected proteins were methylated by PRMT7 in vitro (Fig. 1g). △Np63α served as a negative control (Fig. 1g). The expression of the purified PRMT7 protein was examined by Coomassie blue staining (Fig. 1h). To further test whether MMA sites identified in our MS analysis could indeed be methylated by PRMT7, we mixed purified wild-type (WT) or enzymatic inactive mutant (MT) PRMT7 with synthetic peptides containing the arginine residue being methylated. As shown, PRMT7 (WT), but not PRMT7 (MT), methylated all the synthetic peptides as detected by immunoblotting with anti-MMA antibody (Fig. 1i, top panel). Synthetic peptides from the amino (N)-terminal of histones H3 and H4 served as negative and positive controls, respectively (Fig. 1i, top panel). Meanwhile, PRMT7 did not dimethylate any of the peptides tested (Fig. 1i, middle and bottom panels). The expression of purified PRMT7 (WT) and PRMT7 (MT) proteins was examined by immunoblotting (Fig. 1j). We further demonstrated the occurrence of monomethylation on selected peptides using MALDI-TOF MS analysis (Supplementary Fig. 1c–f). Five arginine methylation sites (R194, 206, 218, 225, and 232) localized in the Arg-Gly Gly (RGG) repeat region of hnRNPA1 were regulated by PRMT7. hnRNPA1 mutants with each (R194K, R206K, R218K, R225K, and R232K) or all five arginine sites replaced by lysine (R/K (7)) were generated. In vitro methylation assay was performed by mixing purified PRMT7 proteins with hnRNPA1 (WT) or mutants. Single site mutants attenuated PRMT7-mediated methylation, and mutants with all five arginine sites replaced had the most dramatic attenuation effect, suggesting that the five arginine sites regulated by PRMT7 in hnRNPA1 can be validated in vitro (Fig. 1k). Taken together, our MS analysis results revealed that PRMT7 regulates the methylation of a large number of proteins, which were experimentally validated in vitro.

### Characterization of PRMT7-mediated methylation

**Fig. 2:**
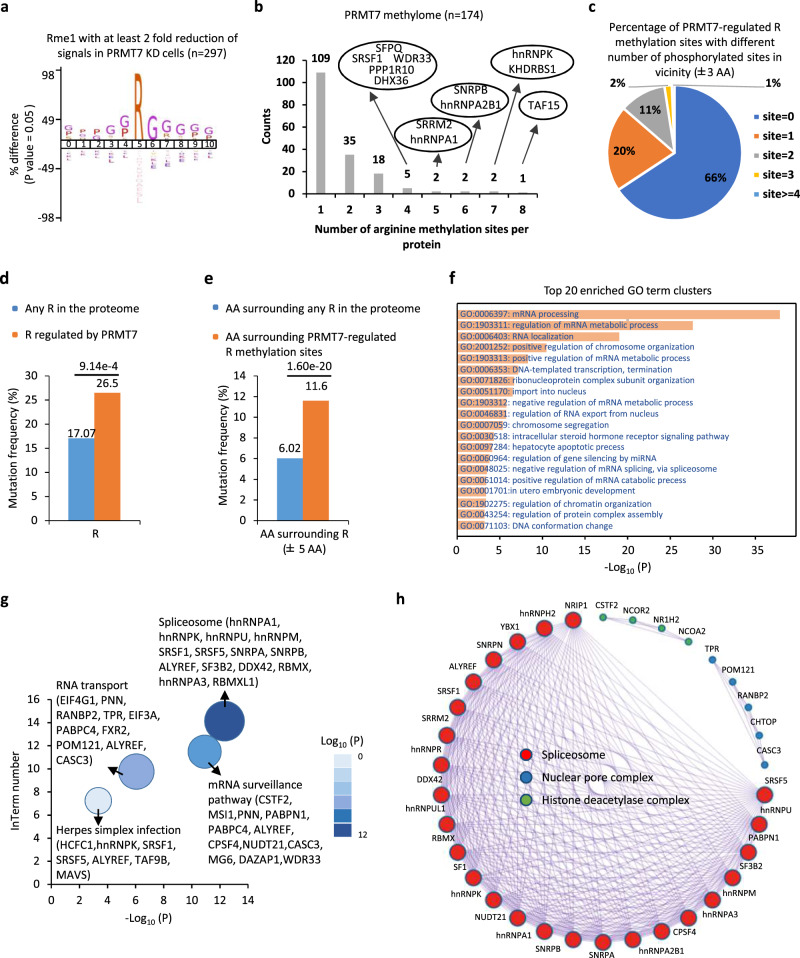
Characterization of PRMT7-regulated arginine methylation sites. **a** Motif analysis was performed for PRMT7-regulated arginine methylation sites using iceLogo^84^. **b** The distribution of proteins in PRMT7 methylome with different number of arginine methylation sites. The proteins with more than two methylation sites were shown in oval. **c** The percentage of PRMT7-regulated arginine methylation sites with different number of phosphorylation sites (*n* = 0, 1, 2, 3 or ≥4) in vicinity (±3 amino acid (AA) window) was shown by pie chart. **d** Rates of somatic mutations at all arginine sites in the proteome or PRMT7-regulated arginine sites in human cancers (*p* = 9.14e−4 by the Fisher’s exact test). **e** Rates of somatic mutations in the vicinity of all arginine sites in the proteome or PRMT7-regulated arginine sites in human cancers (±5 nucleotides) (*p* = 1.6e−20 by the Fisher’s exact test). **f**–**h** Gene ontology (GO) (**f**), KEGG pathway (**g**), and CORUM^47^ (**h**) analysis using Metascape^46^ for PRMT7 methylome. Representative terms from the top 20 enriched GO term clusters were shown.

First, motif analysis results revealed that the most enriched motif was the GAR (glycine and arginine) motif in PRMT7-regulated monomethylated peptides (58.6%), and there were also a large number of PRMT7-regulated monomethylated peptides had the RXR motif (19.9%) as reported previously^38,39^ (Fig. 2a). We then assessed the distribution of PRMT7-regulated arginine methylation sites and found that 37.4% of PRMT7 substrates harbored more than one arginine methylation site regulated by PRMT7 (Fig. 2b and Supplementary Data 1). In particular, eight arginine methylation sites were regulated by PRMT7 in the TAF15 protein (Fig. 2b and Supplementary Data 1).

Arginine methylation sites regulated by PRMT1, PRMT4, and PRMT5 are often found to be adjacent to phosphorylation sites^8^. Similarly, with a seven-amino acid (AA) (±3 AA) window, approximately 34% of the PRMT7-regulated arginine methylation sites had at least one phosphorylation site in proximity (Fig. 2c), which was significantly higher than that of randomly selected arginine sites in the proteome (Supplementary Fig. 2a). A similar observation was reported using an eleven-amino acid window (±5 AA) (Supplementary Fig. 2b, c). The co-occurrence of arginine methylation and phosphorylation was demonstrated on representative PRMT7 substrates (Supplementary Fig. 2d–f). These arginine methylation and phosphorylation sites are localized in the intrinsic disorder region, which is consistent with recent reports implicating arginine methylation in phase separation (Supplementary Fig. 2b–f)^40–44^.

The clinical significance of arginine methylation was underscored by the vulnerability of their sites to cancer mutations^7^. According to the catalogue of somatic mutations in cancer^45^, mutation frequency at and in proximity of PRMT7-regulated arginine methylation sites was significantly higher when compared to that of all arginine sites in the proteome, suggesting that PRMT7-regulated arginine methylation might be functionally important (Figs. 2d and e).

We next examined the biological processes and pathways that PRMT7 methylome was involved in using Metascape^46^. PRMT7 methylome was mainly enriched with gene ontology (GO) terms in RNA biology (mRNA processing, RNA metabolic process, RNA localization, RNA transport, mRNA translation, ribonucleoprotein complex subunit organization and gene silencing by miRNA, and mRNA splicing) and chromosome/chromatin biology (chromosome organization, DNA-templated transcription, chromosome segregation, and DNA conformational change) (Fig. 2f and Supplementary Data 4). Furthermore, the results of the Kyoto Encyclopedia of Genes and Genomes (KEGG) pathway analysis revealed that PRMT7 methylome is implicated in spliceosome, mRNA surveillance pathway, RNA transport, and herpes simplex infection (Fig. 2g and Supplementary Data 4). In particular, a large number of splicing factors, such as hnRNPA1, hnRNPK, hnRNPU, hnRNPM, hnRNPA3, SRSF1, SRSF5, SNRPA, SNRPB, and SF3B2, are methylated by PRMT7, suggesting that PRMT7 regulates RNA splicing. CORUM (the comprehensive resource of mammalian protein complexes database)^47^ was applied to further investigate the interaction network and macromolecular complexes in PRMT7 methylome. Spliceosome, nuclear pore complex, and histone deacetylase complex were the three main complexes found (Fig. 2h).

### Features of arginine methylation mediated by PRMT members

**Fig. 3:**
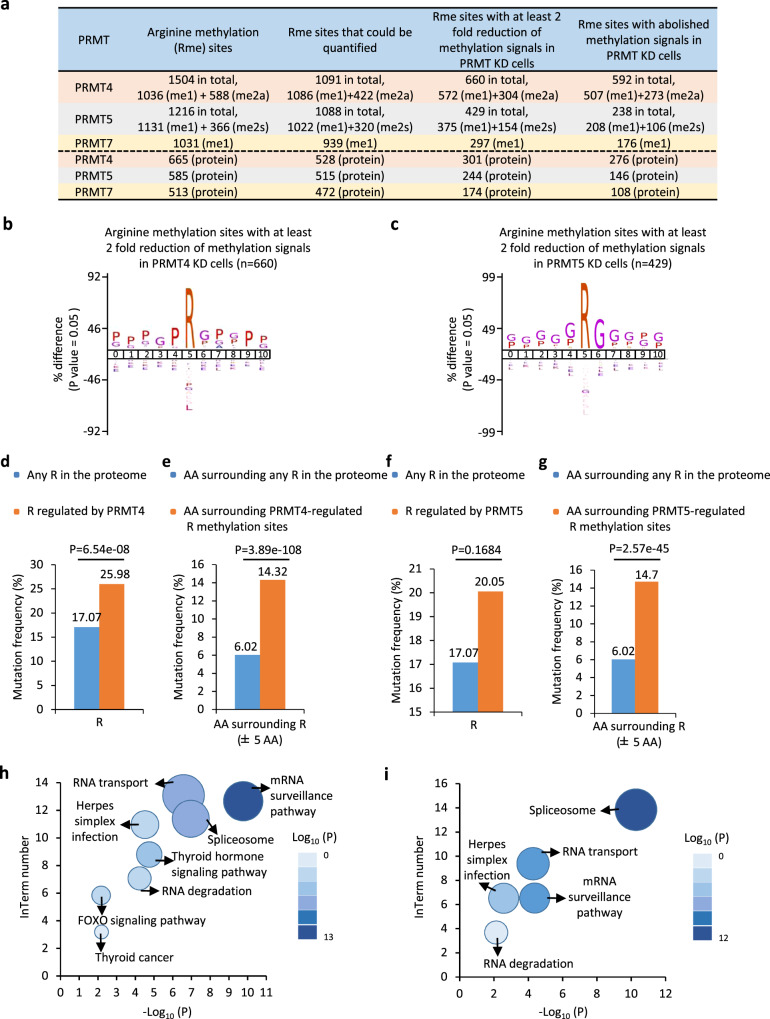
Comparison among PRMT7, 4, and 5-regulated arginine methylation revealed that these three PRMTs all methylate proteins with implications in RNA biology. **a** The number of arginine methylation sites detected (the sum of mono- and di-methylation sites after removing duplicates) (column 2), arginine methylation sites could be quantified (column 3), arginine methylation sites with methylation signals decreased at least two-fold (column 4) or abolished (column 5) in PRMT4-, PRMT5- or PRMT7-knockdown experiments (lane 2, 3, and 4) following mass spectrometry analysis as described in Fig. 1d. The number of proteins encompass all these methylation sites was also shown (bottom three lanes). For comparison, data shown for PRMT7 in Fig. 1e was also included here. **b**, **c** Motif analysis was performed for PRMT4- (**b**) and PRMT5- (**c**) regulated arginine methylation sites using iceLogo^84^. **d**, **f** Rates of somatic mutations at all arginine sites in the proteome and PRMT4- (**d**) or PRMT5- (**f**) regulated methylation sites in human cancers (*p* = 6.54e−08 (**d**) and *p* = 0.1684 (**f**) by the Fisher’s exact test). **e**, **g** Rates of somatic mutations in the vicinity of all arginine sites in the proteome or PRMT4- (**e**) or PRMT5- (**g**) regulated methylation sites in human cancers (±5 nucleotides) (*p* = 3.89e−108 (**e**) and *p* = 2.57e−45 (**g**) by the Fisher’s exact test). **h**, **i** KEGG pathway analysis using Metascape^46^ for PRMT4 (**h**) and PRMT5 (**i**) methylome. Representative terms from the top 20 enriched GO term clusters were shown.

To understand the network of arginine methylation mediated by different types of PRMT, similar approaches used to identify PRMT7-regulated arginine methylation were employed for PRMT4 and PRMT5, which were chosen to represent types I and II PRMTs, respectively. Anti-MMA- and aDMA-specific antibodies were used for immunoenrichment to identify PRMT4-regulated methylation sites, whereas anti-MMA and anti-sDMA-specific antibodies were used for immunoenrichment to identify PRMT5-regulated methylation sites. The knockdown efficiency of PRMT4 and PRMT5 were examined by immunoblotting (vide infra) (Supplementary Fig. 3a, b). A total of 1504 (1036 MMA and 588 aDMA) methylation sites in 665 proteins and 1216 (1,131 MMA and 366 sDMA) methylation sites in 585 proteins were predicted in PRMT4- and PRMT5-knockdown experiments, respectively (Fig. 3a, the second column). Approximately 70% of MMA sites were identified in all three knockdown experiments, supporting the reproducibility of our MS analysis. 660 sites from 301 proteins had at least two-fold reduction in methylation signals, and these 301 proteins were referred to as “PRMT4 methylome” (Fig. 3a, the fourth column and Supplementary Data 5). PRMT5 methylome was similarly defined, with 244 proteins bearing 429 PRMT5-regulated arginine methylation sites (Fig. 3a, the fourth column and Supplementary Data 6). In particular, the methylation of 592 arginine sites from 276 proteins and 238 sites from 146 proteins was completely abolished when PRMT4 and PRMT5 were knocked down, respectively (Fig. 3a, fifth column). The observed change in the levels of arginine methylation was not due to the change in the abundance of the corresponding proteins (Supplementary Fig. 3c, d, Supplementary Data 5 and 6). Known substrates for PRMT4, such as MED12, SMARCC1, and EP300, and for PRMT5, such as FUS, hnRNPA1, and SMN1, were identified (Supplementary Data 5 and 6).

We next characterized PRMT4- and PRMT5-mediated arginine methylation sites and compared them with PRMT7. Results from motif analysis revealed a consensus GAR (glycine and arginine) motif for PRMT5-regulated arginine methylation, whereas a proline-enriched motif was found for PRMT4-regulated arginine methylation, which was consistent with previous reports (Fig. 3b and c)^7,8^. A large portion of PRMT4 or PRMT5 substrates harbored more than one regulated arginine methylation site (Supplementary Fig. 3e, f, Supplementary Data 5 and 6). In particular, there were sixteen arginine methylation sites regulated by PRMT4 in KMT2D and DERPTC proteins, and nine were regulated by PRMT5 in EIF4G1 protein (Supplementary Fig. 3e, f, Supplementary Data 5 and 6). Similar to PRMT7, PRMT4-, and PRMT5-regulated arginine methylation often co-occurred with phosphorylation (Supplementary Fig. 3g–j) and their methylation sites and sequences in the vicinity were vulnerable to cancer mutations (Figs. 3d–g).

The biological processes and pathways PRMT4 and PRMT5 methylomes were also analyzed and compared to that of PRMT7 methylome. Similar to PRMT7, both PRMT4 and PRMT5 methylomes are mainly enriched with GO terms in RNA biology (mRNA processing, RNA localization, and RNA transport) and chromosome/chromatin biology (Supplementary Fig. 3k, l). Meanwhile, PRMT4, 5, and 7 methylomes were implicated in unique biological processes. For instance, PRMT4 uniquely methylated a cohort of proteins with implications in the regulation of signal transduction by the p53 class mediator; PRMT5 uniquely methylated a cohort of proteins with implications in regulation of the cell cycle process; and PRMT7 uniquely methylated a cohort of proteins with implications in the regulation of utero-embryonic development (Supplementary Fig. 3k, l, and Fig. 2f). KEGG pathway analysis results revealed that the top four most enriched pathways for PRMT4 and PRMT5 methylomes were spliceosome, mRNA surveillance pathway, RNA transport, and herpes simplex infection, which were similar to those for PRMT7 methylome (Figs. 3h, i).

### PRMT4, 5, and 7 co-regulate a subset of alternative splicing events

**Fig. 4:**
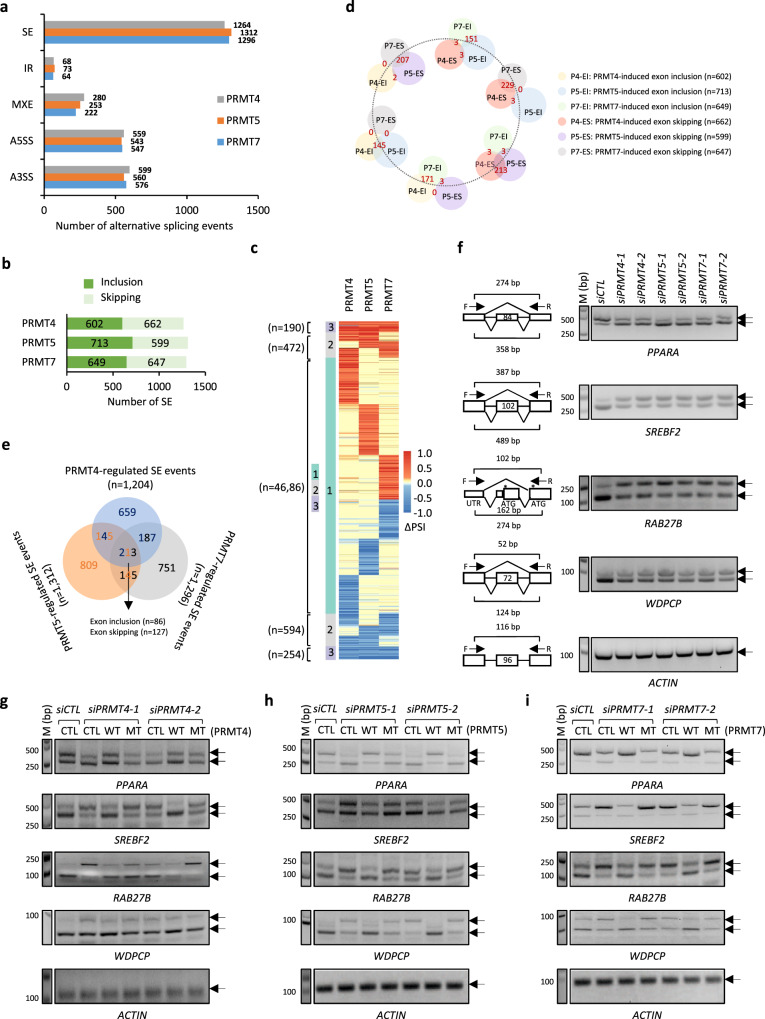
RNA-seq profiling revealed that PRMT4, 5, and 7 exhibited a global impact on RNA alternative splicing. **a** HEK293 cells transfected with *siCTL* or *siPRMT4*, *siPRMT5* or *siPRMT7* were subjected to RNA-seq analysis, and the number of alternative splicing events regulated is shown (|ΔPSI| ≥ 0.5). Cassette exons (SE); intron retain (IR); mutually exclusive exons (MXE); alternative 5′ splice sites (5′ ASS) and alternative 3′ splice sites (3′ ASS). **b** The number of exon inclusion (EI) and skipping (ES) induced is shown. **c** All alternative splicing events regulated by PRMT4, 5, and 7 were classified into three categories, events regulated by one (light green), any two out of three (light black) or all three (light purple) PRMTs. **d** Overlap between EI and ES induced by PRMT4, 5, or 7 is shown. **e** Overlap among PRMT4-, 5-, and 7-regulated cassette exons is shown. **f** HEK293 cells transfected with *siCTL* or two individual *siPRMT4* (*siPRMT4-1* and *siPRMT4-2*), *siPRMT5* (*siPRMT5-1* and *siPRMT5-2*), or *siPRMT7* (*siPRMT7-1* and *siPRMT7-2*) were subjected to analysis of the expression of both short and long isoforms of representative genes as indicated. The length of the alternatively spliced exon, as well as the expected length of the PCR products, was shown as indicated. DNA marker (M) was included on the left (bp: base pair). The position of the cassette exon in each gene was as follows: *PPARA* (NM_005036, exon3); *SREBF2* (NR_103834, exon18); *RAB27B* (NM_001375327, exon3); *WDPCP* (NM_015910, exon6). F: forward primer; R: reverse primer. The translation start sites (ATG) of the two isoforms of *RAB27B* were indicated by asterisks. *n* = 3 biological replicates and representative data are shown. **g**–**i** HEK293 cells transfected with *siCTL* or *siPRMT4* (**g**), *siPRMT5* (**h**), or *siPRMT7* (**i**) in the presence or absence of wild-type (WT) or enzymatic dead mutant (MT) PRMT4 (**g**), PRMT5 (**h**), or PRMT7 (**i**) were subjected to alternative splicing analysis as described in (**f**). Source data are provided as a Source data file.

The enrichment of proteins in RNA biology, especially in spliceosome, for PRMT4, 5, and 7 methylomes prompted us to examine their functional role in RNA splicing. Following the pipeline, as depicted in Supplementary Fig. 4a, we analyzed the alternative splicing events regulated by PRMT4, 5, and 7 in RNA-seq and found that their inhibition all caused significant changes in many alternative splicing events, including cassette exons (SE), intron retention (IR), mutually exclusive exons (MXE), alternative 5′ splice sites (5′ ASS), and alternative 3′ splice sites (3′ ASS) (Fig. 4a). Specifically, the inhibition of PRMT4, 5, and 7 led to significant changes in 2770, 2741, and 2705 alternative splicing events, respectively (|ΔPSI| ≥ 0.5) (Fig. 4a). For cassette exons, all three seemed to have no preference for inclusion or skipping (Fig. 4b).

We then compared the alternative splicing events regulated by PRMT4, 5, and 7 and found that a large set of alternative splicing events is uniquely regulated by each PRMT, and a smaller set of alternative splicing events was regulated by two of three PRMTs or all PRMTs (Fig. 4c). Of the 444 alternative splicing events commonly regulated by all three PRMTs, 213 were SE (Fig. 4c). When one event was regulated by any of the three PRMTs in one direction, then, it is hardly regulated by the other two in the opposite direction (i.e., red- and blue-colored splicing events were almost not co-existing) (Fig. 4c). For instance, only 2 PRMT4-induced exon inclusion events were skipped by PRMT5 and none by PRMT7, and only 3 PRMT4-induced exon skipping events were included by PRMT5 or PRMT7 (Fig. 4d). In particular, for the 213 SE that were commonly regulated by all three PRMTs, they were regulated in the same direction without exception, with the inclusion of 86 and skipping of 127 events being induced by all three PRMTs (Fig. 4e).

We then validated these PRMT4, 5, and 7 co-regulated cassette exons by RT-PCR analysis. Of the ten randomly selected events, nine were validated (Fig. 4f and Supplementary Fig. 4b–d). The knockdown efficiency of PRMT4, 5, and 7 was examined by immunoblotting analysis (Supplementary Fig. 4e). Interestingly, the knockdown of PRMT7, in addition to double knockdown of PRMT4 and PRMT5, further induced changes in alternative splicing, suggesting that PRMT7-mediated monomethylation might be essential in regulation of PRMT4, 5, and 7 co-regulated cassette exons (Supplementary Fig. 4f–h).

To examine whether PRMT4, 5, and 7 regulation of alternative splicing is dependent on their enzymatic activity, we performed rescue experiments with wild-type (WT) or enzymatic inactive mutant (MT) enzymes^38,48–50^. The corresponding wild-type, but not the enzymatic-inactive mutant, rescued the change in alternative splicing caused by PRMT4, 5, or 7 knockdown for the selected genes (Figs. 4g–i and Supplementary Fig. 4i–k). We also tested the effects of PRMT1 and found that its knockdown similarly led to significant changes in the alternative splicing events tested (Supplementary Fig. 4l, m). The expression of wild-type and enzymatically inactive mutants was examined by immunoblotting analysis (Supplementary Fig. 4n). Taken together, PRMT4, 5, and 7 change the alternative splicing of many genes, and in particular, they tend to regulate alternative splicing in the same direction.

### hnRNPA1 methylation regulates PRMT4, 5, and 7 co-regulated events

**Fig. 5:**
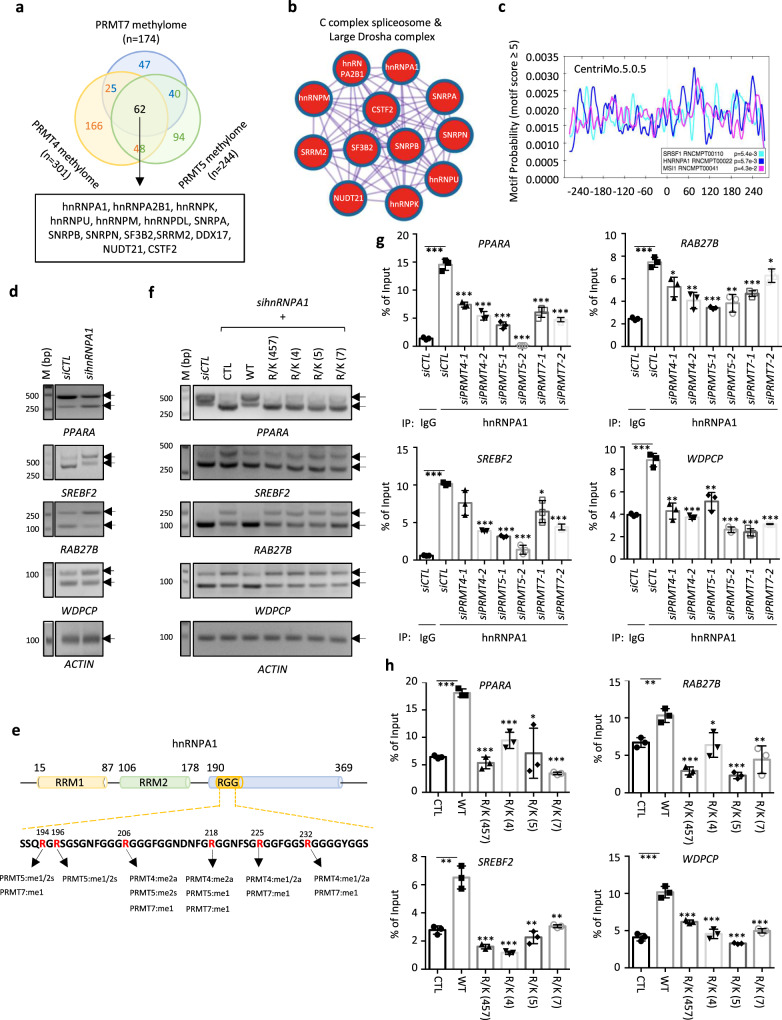
PRMT4-, 5-, and 7-mediated hnRNPA1 methylation was involved in the regulation of alternative splicing events co-regulated by the three PRMTs. **a** Overlap among PRMT4, 5, and 7 methylome is shown. Splicing factors among substrates commonly regulated by PRMT4, 5, and 7 are listed. **b** Metascape CORUM^46,47^ analysis was performed for substrates commonly regulated by PRMT4, 5, and 7. **c** Motif analysis was performed for cassette exons commonly regulated by PRMT4, 5, and 7 using CentriMo (v5.0.5)^51^. **d** HEK293 cells transfected with *siCTL* or *sihnRNPA1* were subjected to alternative splicing analysis as described in Fig. 4f. **e** Schematic representation of the domain architecture of hnRNPA1. Arginine (R) sites methylated by PRMT4, 5, and/or 7 in the RGG domain were highlighted in red. The PRMTs that methylated each arginine and the methylation status were indicated at the bottom. (RRM: RNA recognition motif; RGG: arginine-glycine-glycine). **f** HEK293 cells transfected with *siCTL* or *sihnRNPA1* in the presence or absence of wild type (WT) or methylation mutants including R/K (4), R/K (5), R/K (7), and R/K (457) were subjected to alternative splicing analysis as described in Fig. 4f. **g** HEK293 cells transfected with *siCTL* or *siPRMT4*, *5*, or *7* were subjected to RNA-IP assay using control IgG or anti-hnRNPA1 antibody. *n* = 3 biological replicates (mean ± SEM, *P < 0.05, **P < 0.01, ***P < 0.001 by unpaired Student *t*-test, two-tailed). **h** HEK293 cells transfected with empty vector (CTL) or Flag-tagged, wild-type (WT) or methylation mutants as described in (**f**) were subjected to RNA-IP assay using anti-Flag antibody. *n* = 3 biological replicates (mean ± SEM, *P < 0.05, **P < 0.01, ***P < 0.001 by unpaired Student *t*-test, two-tailed). Source data are provided as a Source Data file.

The observation that PRMT4, 5, and 7 co-regulate a subset of alternative splicing events in an enzymatic-dependent manner and that PRMT4, 5, and 7 methylomes share a large number of RNA splicing factors prompted us to examine whether arginine methylation on these RNA splicing factors is involved in the regulation of alternative splicing events that these three PRMTs co-regulate. To this end, PRMT4, 5, and 7 methylomes were compared, and 62 proteins were found to be commonly regulated by these three PRMTs (Fig. 5a and Supplementary Data 7). Importantly, these 62 proteins were highly enriched with splicing factors, particularly components of the C complex spliceosome and the large Drosha complex (Fig. 5b).

To assess whether any of the commonly regulated substrates could partly account for the commonly regulated alternative splicing events by PRMT4, 5, and 7, we first analyzed the RNA sequences upstream of the 3′ splice site by CentriMo^51^. CentriMo was designed for local motif enrichment analysis, which identifies known or user-provided motifs that show a significant preference for particular locations in the input sequences. Binding motifs for several splicing factors were enriched, including hnRNPA1, which was a substrate commonly regulated by PRMT4, 5, and 7 (Fig. 5c). hnRNPA1 is one of the most abundant and ubiquitously expressed nuclear proteins and plays a major role in mRNA biogenesis. hnRNPA1 indeed exhibited similar effects as PRMT4, 5, and 7 on the commonly regulated alternative splicing events tested (Fig. 5d). The knockdown efficiency of hnRNPA1 was examined using RT-qPCR and immunoblotting analysis (Supplementary Fig. 5a, b).

We then focused on investigating whether and how PRMT4-, 5-, and 7-mediated arginine methylation on hnRNPA1 is involved in the regulation of these alternative splicing events commonly regulated by PRMT4, 5, and 7. The flexible, glycine-rich carboxyl (C)-terminal region consisting of Arg-Gly-Gly (RGG) tripeptide repeats provides hnRNPA1 with both protein- and RNA-binding capabilities (Fig. 5e). All six arginine residues within the RGG repeat region were recovered in our MS analysis, among which R206me2a, R218me2a, R225me1/2a, and R232me1/2a were regulated by PRMT4; R194me1/2s, R196me1/2s, R206me2s, and R218me1 were regulated by PRMT5; and R194me1, R206me1, R218me1, R225me1, and R232me1 were regulated by PRMT7 (Fig. 5e).

Next, we examined whether these identified arginine methylation sites are involved in the regulation of common alternative splicing events by PRMT4, 5, and 7. We first generated single site mutants by replacing arginine (R) with lysine (K), including R194K, R196K, R206K, R218K, R225K, and R232K. Each of these single site mutants attenuated hnRNPA1 function in alternative splicing regulation, albeit to different extents (Supplementary Fig. 5c, d). The expression of hnRNPA1 mutants was examined by immunoblotting analysis (Supplementary Fig. 5e). Furthermore, arginine residues that were methylated by PRMT4 (R/K (4)), PRMT5 (R/K (5)), PRMT7 (R/K (7)), or all three PRMTs (R/K (457)) were replaced with lysine. hnRNPA1 mutants did not rescue alternative splicing changes caused by hnRNPA1 knockdown as efficiently as wild-type hnRNPA1 (Fig. 5f and Supplementary Fig. 5f). The expression of hnRNPA1 mutants was examined by immunoblotting analysis (Supplementary Fig. 5g).

The PRMT-mediated arginine methylation on a number of proteins can regulate their direct interaction with RNA^52–54^. We then first investigated whether PRMT4, 5, and 7 are involved in hnRNPA1 binding with pre-mRNAs. The knockdown of PRMT4, 5, or 7 significantly attenuated hnRNPA1 binding with the pre-mRNAs of co-regulated genes (Fig. 5g). The IP efficiency of hnRNPA1 was shown to be equal between different experimental conditions as examined by immunoblotting analysis (Supplementary Fig. 5h). Interestingly, the knockdown of PRMT7, in addition to double knockdown of PRMT4 and PRMT5, further attenuated hnRNPA1 binding with the pre-mRNAs of co-regulated genes, which was consistent with the observation that PRMT7 knockdown further induced changes in alternative splicing (Supplementary Figs. 5i, j, and 4f–h).

Then, we compared the RNA binding affinity between the wild-type and mutant hnRNPA1 using RNA-IP experiments. We revealed that all mutants exhibited a much weaker binding with the pre-mRNA of selected genes than wild-type hnRNPA1 (Fig. 5h and Supplementary Fig. 5k). The IP efficiency of hnRNPA1 proteins, both wild-type and mutant, was examined by immunoblotting analysis (Supplementary Fig. 5l, m). It should be noted that the decrease in binding with pre-mRNA was not due to the change in subcellular localization of hnRNPA1 mutants as they either had no impact on subcellular localization or exhibited greater nuclear localization (Supplementary Fig. 5n). The R/K (457) mutant showed no significant effect on hnRNPA1 interaction with other splicing factors complexed with hnRNPA1 and methylated by PRMT7, such as hnRNPA2B1, hnRNPK, hnRNPU, and hnRNPM. This suggests that arginine methylation directly regulates hnRNPA1 binding with RNA rather than binding with other splicing factors to regulate alternative splicing (Supplementary Fig. 5o). Taken together, the PRMT4, 5, and 7-mediated arginine methylation on splicing factor hnRNPA1 is involved in the regulation of alternative splicing events commonly regulated by all three PRMTs.

### PRMT4, 5, 7, and hnRNPA1 methylation are highly enriched in cancers

**Fig. 6:**
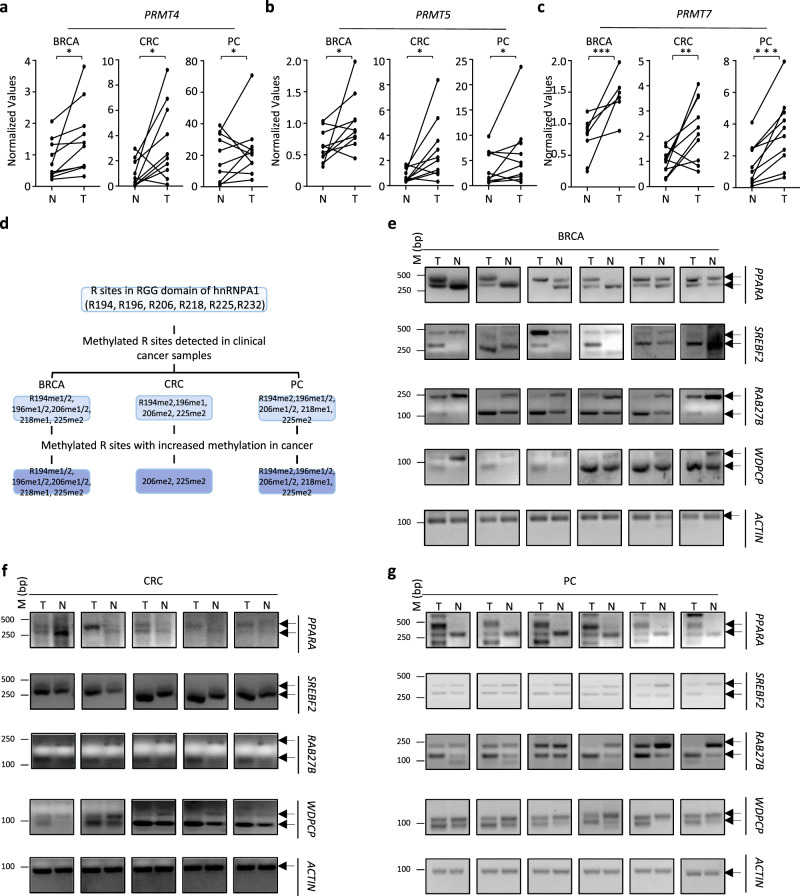
PRMT4, 5, 7, and hnRNPA1 arginine methylation were over-presented in multiple types of cancers. **a**–**c** Clinical samples of breast cancer (BRCA) (*n* = 10), colorectal cancer (CRC) (*n* = 10) and prostate cancer (PC) (*n* = 10) were subjected to RNA extraction and RT-qPCR analysis to examine the expression of *PRMT4* (**a**), *PRMT5* (**b**), and *PRMT7* (**c**). Pearson correlation coefficient of the expression of *PRMT4*, *PRMT5*, and *PRMT7* between normal and tumor samples is 0.60, −0.50, and −0.17 in BRCA, 0.12, 0.21, and −0.05 in CRC, and 0.48, 0.74, and 0.69 in PC, respectively. (*P < 0.05, **P < 0.01, ***P < 0.001 by paired Student *t*-test, two-tailed). **d** Arginine methylation sites in RGG domain of hnRNPA1 protein that could be detected and quantified in clinical samples of BRCA, CRC, and PC were shown. **e**–**g** Clinical samples of BRCA (**e**), CRC (**f**), and PC (**g**) were subjected to alternative splicing analysis as described in Fig. 4f. Source data are provided as a Source data file.

Alterations in PRMT expression and gene alternative splicing have implicated in disease development and progression, particularly in cancer^11,18,55,56^. To examine whether the expression of PRMT4, 5, and 7, the methylation of hnRNPA1, or the alternative splicing events they regulate are altered in cancers, we first examined the expression of PRMT4, 5, and 7 in clinical tissue samples from several types of cancers, including breast cancer (BRCA), colorectal cancer (CRC), and prostate cancer (PC). The expression of PRMT4, 5, and 7 was significantly higher in tumor tissues than in adjacent normal tissues (Figs. 6a–c), which is consistent with previous reports^10,11^. Next, we examined the arginine methylation of hnRNPA1 in BRCA, CRC, and PC samples. Four pairs of normal and tumor tissues for each cancer type were subjected to tandem mass tag (TMT) labeling and quantitative MS analysis followed by arginine methylation search. Arginine methylation in the RGG domain of hnRNPA1 was often significantly higher than that in normal samples in all three types of cancers (Fig. 6d and Supplementary Data 8). Along with high levels of methylation of PRMT4, PRMT5, PRMT7, and hnRNPA1 in tumor samples, changes in gene alternative splicing were observed, such as those for *PPARA*, *SREBF2*, *RAB27B*, and *WDPCP* in BRCA, CRC, and PC samples (Fig. 6e–g and Supplementary Fig. 6a–c).

### PRMT4, 5, 7, and hnRNPA1 methylation are required for the growth of cancer cells

**Fig. 7:**
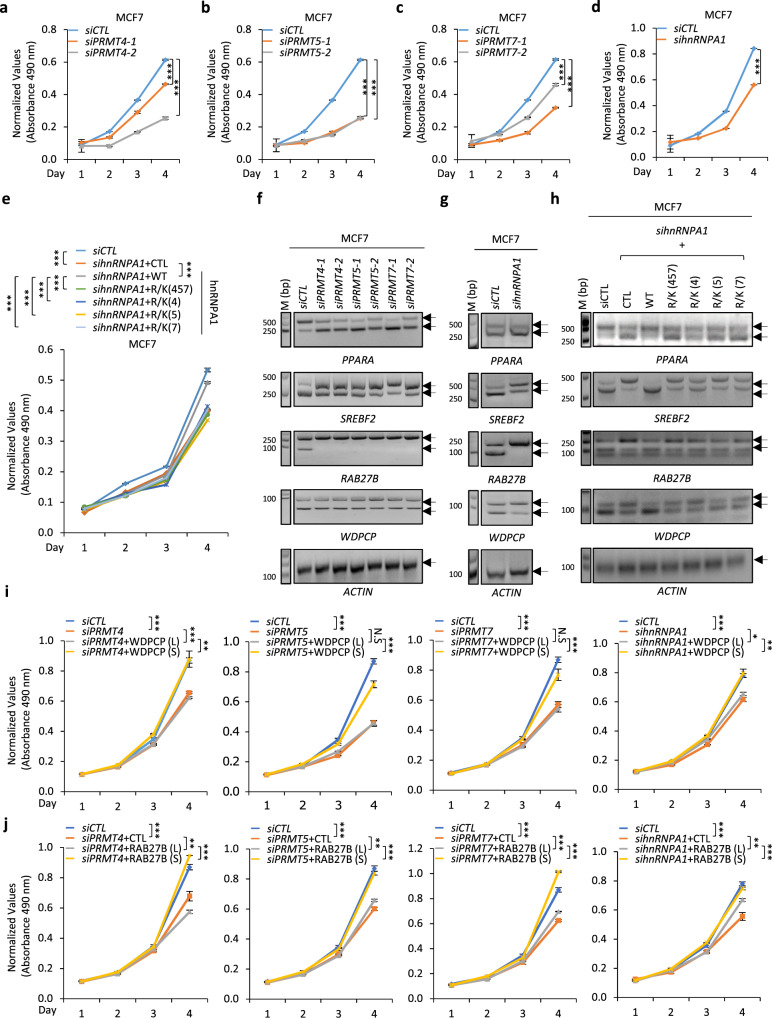
PRMT4, 5, 7, and hnRNPA1 arginine methylation were required for the growth of multiple types of cancer cells. **a**–**c**, **f** MCF7 cells were transfected with *siCTL* or *siPRMT4*, *5*, or *7* followed by cell proliferation assay (**a**–**c**) and alternative splicing analysis (**f**). *n* = 3 biological replicates for cell proliferation assay (mean ± SD, ***P < 0.001, day 4 by unpaired Student *t*-test, two-tailed). *n* = 3 biological replicates for alternative splicing analysis and representative data are shown. **d**, **g** MCF7 cells were transfected with *siCTL* or *sihnRNPA1* followed by cell proliferation assay (**d**) or alternative splicing analysis (**g**). *n* = 3 biological replicates for cell proliferation assay (mean ± SD, ***P < 0.001, day 4 by unpaired Student *t*-test, two-tailed). **e**, **h** MCF7 cells transfected with *siCTL* or *sihnRNPA1* in the presence or absence of wild type (WT) or methylation deficient mutants including R/K (4), R/K (5), R/K (7), and R/K (457) were subjected to cell proliferation assay (**e**) or alternative splicing analysis (**h**). *n* = 3 biological replicates for cell proliferation assay (mean ± SD, ***P < 0.001, day 4 by unpaired Student *t*-test, two-tailed). **i**, **j** MCF7 cells were transfected with *siCTL* or *siPRMT4*, *siPRMT5*, *siPRMT7*, or *sihnRNPA1* in the presence or absence of Flag-tagged WDPCP (**i**) or RAB27B (**j**), both short (S) and long (L) isoforms, followed by cell proliferation assay. *n* = 3 biological replicates (mean ± SD, *P < 0.05, **P < 0.01, ***P < 0.001, N.S: non-significant, day 4 by unpaired Student *t*-test, two-tailed). Source data are provided as a Source data file.

The upregulation of PRMT4, PRMT5, PRMT7, and hnRNPA1 arginine methylation, as well as the altered alternative splicing in clinical BRCA, CRC, and PC tissue samples, prompted us to examine whether these three PRMTs and hnRNPA1 methylation are functionally important in regulating the growth of these cancer cells. The knockdown of PRMT4, PRMT5, PRMT7, or hnRNPA1 led to slower proliferation rate in MCF7 cells (Figs. 7a–d). The critical roles of PRMT4, PRMT5, PRMT7, and hnRNPA1 in cell growth were also demonstrated in HCT 116 cells (Supplementary Fig. 7a, b), a colon cancer cell line, and LNCaP (Supplementary Fig. 7c, d), a prostate cancer cell line. Similar observations were found in the colony formation assay (Supplementary Fig. 7e–p).

Thus, we assessed the potential role of arginine methylation sites in regulating cell growth and found that methylation mutants did not as efficiently rescue cell growth defects following the knockdown of hnRNPA1 as wild-type hnRNPA1 as evidenced by cell proliferation and colony formation assays (Fig. 7e and Supplementary Fig. 7q–r). The knockdown of PRMT4, PRMT5, PRMT7, and hnRNPA1 were associated with changes in gene alternative splicing, such as that of *PPARA*, *SREBF2*, *RAB27B*, and *WDPCP* (Figs. 7f, g, and Supplementary Fig. 7s, t), in a manner dependent on arginine sites methylated by PRMT4, 5, or 7 (Fig. 7h and Supplementary Fig. 7u). We then evaluated whether PRMT4, PRMT5 and PRMT7-regulated alternative splicing events are involved in cancer cell growth. Both short isoforms of WDPCP and RAB27B, which are induced by PRMT4, 5, and 7, promoted the proliferation of MCF7 cells, whereas the long isoform did so to a much lesser extent or even exhibited an opposite effect, thus linking aberrant splicing to cancer cell growth (Supplementary Fig. 7v–y). The expression of WDPCP and RAB27B isoforms was examined by immunoblotting (Supplementary Fig. 7z). It should be noted that the long isoform of RAB27B might also utilize the translation start codon (ATG) of the short isoform, resulting in two distinct bands being detected (Supplementary Fig. 7z). Furthermore, both short isoforms of WDPCP and RAB27B rescued the proliferation defects caused by PRMT4, PRMT5, PRMT7, and hnRNPA1 knockdown in MCF7 cells, whereas the long isoform did so to a much lesser extent (Figs. 7i and j). Taken together, PRMT4, PRMT5, PRMT7 and potentially hnRNPA1 arginine methylation were required for the growth of multiple types of cancer cells.

### Pharmacological inhibition of PRMT4, 5, and 7 suppresses cancer cell

**Fig. 8:**
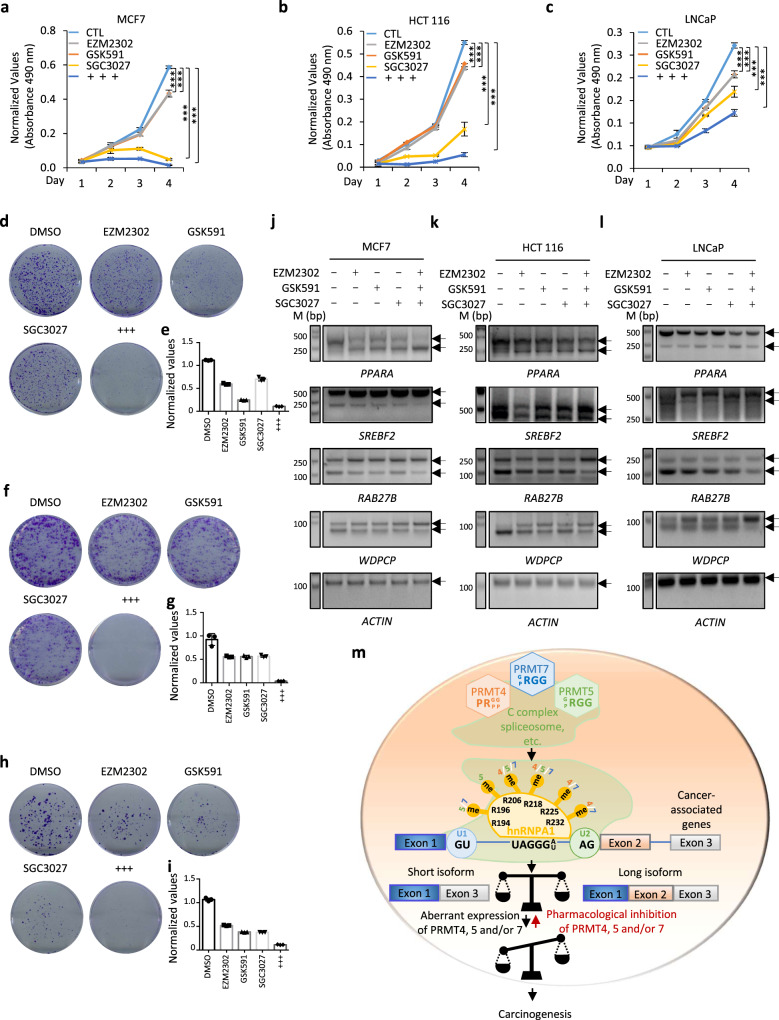
Pharmacological inhibition of PRMT4, 5, and 7 alters RNA alternative splicing and suppresses cancer cell growth. **a**–**c**, **d**, **f**, **h** MCF7 (**a**, **d**), HCT 116 (**b**, **f**), or LNCaP (**c**, **h**) cells were treated with EZM2302 (10 μM), GSK591 (10 μM), or SGC3027 (10 μM) alone or in combination (+++) for duration as indicated followed by proliferation assay (**a**–**c**) or colony formation assay (**d**, **f**, **h**). *n* = 3 biological replicates for cell proliferation assay (mean ± SD, **P < 0.01, ***P < 0.001, day 4 by unpaired Student *t*-test, two-tailed). **e**, **g**, **i** Quantification of the crystal violet dye as shown in (**d**), (**f**), and (**h**). Absorbance was measured three time. **j**–**l** MCF7 (**j**), HCT 116 (**k**), or LNCaP (**l**) cells were treated with EZM2302 (10 μM), GSK591 (10 μM), or SGC3027 (10 μM) alone or in combination for 24 h as indicated followed by alternative splicing analysis. *n* = 2 biological replicates and representative data are shown. **m** A working model of PRMT4-, 5-, and 7-mediated arginine methylation in alternative splicing regulation and cancer cell growth. Global profiling of PRMT4-, 5-, and 7-mediated arginine methylation revealed that PRMT4-, 5-, and 7-methylome shared a group of proteins with implications in mRNA splicing, and RNA-seq analysis confirmed the global impact of these three PRMTs on gene alternative splicing. Exemplified by hnRNPA1, a critical regulator of gene alternative splicing, PRMT4-, 5-, and 7-mediated arginine methylation was shown to be involved in hnRNPA1 binding with pre-mRNA and, therefore, the regulation of those alternative splicing events commonly regulated by PRMT4, 5, and 7. In cancers, such as breast, colorectal, and prostate cancer, PRMT4, 5, and 7 were found to be overexpressed, leading to elevated levels of arginine methylation on their substrates including hnRNPA1, and therefore aberrant alternative splicing changes, which potentially drive cancer cell growth. Pharmacological inhibition of PRMT4, 5, and 7 exhibited great potential in suppressing the growth of cancer cells. Source data are provided as a Source data file.

The requirement of PRMT4, 5, and 7 for the cell growth of breast, colon, and prostate cancer suggests that their pharmacological inhibition might represent a target to suppress the growth of these cancer cells. To confirm this, MCF7, HCT116, and LNCaP cells were treated with EZM2302 (a PRMT4-specific inhibitor)^57^, GSK591 (a PRMT5-specific inhibitor)^55,58^, or SGC3027 (a PRMT7-specific inhibitor)^31^ or their combination. As expected, EZM2302 treatment significantly decreased both levels of MMA and ADMA; GSK591 treatment significantly decreased both levels of MMA and SDMA; and SGC3027 only affected MMA levels. These results support the specificity of these inhibitors (Supplementary Fig. 8a–c). EZM2302, GSK591, or SGC3027 treatment alone significantly inhibited the growth of MCF7, HCT116, and LNCaP cells, and their co-treatment exhibited a higher inhibitory effect (Figs. 8a–c). Similar observations were found in the colony formation assay (Fig. 8d–i).

Next, we evaluated whether EZM2302, GSK591, and SGC3027 treatment affected the PRMT4, 5, and 7-regulated alternative splicing. Our data indicate that EZM2302, GSK591, or SGC3027 treatment alone changed alternative splicing events commonly regulated by PRMT4, 5, and 7, and their co-treatment exhibited more drastic effects in all three cell lines, namely MCF7, HCT 116, and LNCaP (Figs. 8j–l and Supplementary Fig. 8d–f). The inhibition of PRMT, in general, had no significant effect on the total RNA levels (Supplementary Fig. 8g). Taken together, the pharmacological inhibition of PRMT4, 5, and 7 was effective in suppressing the growth of cancer cells, such as MCF7, HCT 116, and LNCaP, which was associated with altered gene splicing.

## Discussion

Protein arginine methylation is common PTM, which does not alter the amino acid charge but instead increases the bulkiness and hydrophobicity of amino acids, thus altering the higher-order protein structure and protein–nucleic acid and protein–protein interactions. Arginine methylation is catalyzed by the PRMT family, which is classified into three types (I, II, and III), according to the final methylarginine products generated. Proteomic profiling of the substrates of the members in the PRMT protein family, particularly PRMT1, PRMT4, and PRMT5, has been reported recently^6,7,59^. However, the full scope of arginine methylation catalyzed by the only type III PRMT, PRMT7, and its interaction with type I and II PRMTs remain unknown. In this study, we employed a high-resolution MS approach to globally profile PRMT4-, 5-, and 7-mediated methylation. We revealed that PRMT4-, PRMT5-, and PRMT7-methylome shared a group of proteins implicated in mRNA splicing and co-regulated a cohort of alternative splicing events. The PRMT4, 5, and 7-mediated arginine methylation of the splicing factor hnRNPA1 was involved in hnRNPA1 binding with pre-mRNA and the regulation of alternative splicing events commonly regulated by these three PRMTs. In breast, colorectal, and prostate cancers, PRMT4, 5, and 7 were upregulated and associated with high levels of arginine methylation on their-regulated methylome, such as hnRNPA1, and aberrant gene alternative splicing, thereby potentially driving cancer cell growth (Fig. 8m).

Our MS analysis recovered mono-methylation at 297 arginine sites in 174 proteins that were decreased in response to PRMT7 knockdown. The detailed characterization of these PRMT7-regulated mono-methylation revealed several features: PRMT7 preferentially methylates a consensus GAR (glycine and arginine) motif; PRMT7 often methylates multiple arginine sites in a given protein; phosphorylation sites are often found in the vicinity of PRMT7-regulated arginine methylation sites; PRMT7-regulated methylation sites and sequences in the vicinity are vulnerable to mutations in cancers. Previous studies have reported that PRMT7 has distinct substrate specificity for RXR motifs surrounded by basic residues based on histone substrates^38,39^. In our study, the antibodies used for monomethylation enrichment were a mixture of two antibodies, which were generated using a library of synthetic monomethylarginine peptides (XXXXXXXR*XXXXXX) or R*GG motif (XXXXXXXR*GGXXX) as antigens (see “Methods”), and should have been able to recognize the RGG motif, as well as others, such as the RXR motif. Indeed, approximately 58.6% of the monomethylated peptides responsive to PRMT7 knockdown possessed the RGG motif, and approximately 19.9% had the RXR motif. It should be noted that, Szewczyk et al. recently identified 581 MMA sites from 292 proteins in HCT116 cells. Of these MMA sites, 308 were similarly found in HEK293 cells in this study, thus highlighting the cell-type specificity of arginine methylation^31^. Furthermore, they revealed that 29 methylated peptides from 24 unique proteins are PRMT7-dependent under a more stringent cutoff (FC > 10) compared with ours (FC > 2)^31^. When we re-analyzed their data using a cut-off of FC > 2, we found that 127 MMA sites from 67 proteins are dependent on PRMT7 and that 32 MMA sites are shared by both studies. Of the five arginine residues in hnRNPA1 we identified to be dependent on PRMT7, four (i.e, R194, 206, 218, and 225) were also identified by Szewczyk et al.^31^.

To gain insights into the biological function of PRMT7, we analyzed the proteins in PRMT7 methylome and found that PRMT7 regulates the methylation of a large cohort of proteins with implications in a plethora of biological pathways, including spliceosome, RNA transport, mRNA surveillance pathway, and herpes simplex infection. In particular, a number of splicing factors were methylated by PRMT7, suggesting its putative function in RNA splicing. In addition, the PRMT7 methylome included proteins with implications in chromosome organization, chromosome segregation, chromatin organization, circadian rhythm, and utero embryonic development, which should be investigated further. Comparison among the PRMT4, PRMT5, and PRMT7 methylomes revealed that the substrates of PRMT4 and PRMT5 were also implicated in pathways similar to PRMT7, such as spliceosome, RNA transport, mRNA surveillance pathway, and herpes simplex infection, suggesting that proteins in these pathways are hotspots for PRMT targets. Despite the involvement of the substrates of these three PRMTs in the same pathways, the majority of the substrates were unique for each PRMT. In addition, PRMT4 methylome were found to be implicated in hormone signaling pathway and cancer development, which is consistent with its well-studied function in cancer^60–63^. The effects observed following PRMT7 knockdown might be partly attributed to PRMT5 as PRMT5-mediated methylation is distributive and PRMT7-mediated MMA might affect the PRMT5-mediated SDMA modification^64^.

The enrichment of spliceosome proteins in PRMT4, 5, and 7 methylomes prompted us to examine their global effects on RNA splicing using RNA-seq analysis. PRMT5 has been well-documented to play a critical role in splicing in both mouse and human^55,56,65–67^. PRMT4 and PRMT7 have been linked to splicing due to their ability to directly methylate key proteins in the splicing machinery^68,69^. Cheng et al. have reported that splicing factors, including CA150, SAP49, SmB, and U1C, are methylated by PRMT4 and have demonstrated the function of PRMT4 in splicing using an exogenous splicing reporter and endogenous *CD44* gene^69^. R20me1 of CA150 (TCERG1) and multiple methylation sites in Sm B/B′ (SNRPB), including R94me1, R108me1/2, R112me1/2, R132me1/2, R147me1/2, R172me1/2, R181me1/2, R236me1/2, and R239me1, were dependent on PRMT4 in our dataset (FC > 2)^69^. Gonsalvez et al. have reported that three of the seven Sm proteins, i.e., B/B′, D1, and D3, are symmetrically dimethylated by PRMT7 and that the Sm protein SDMA modification is required for snRNP biogenesis, thus indicating a potential role of PRMT7 in splicing^68^. Multiple methylation sites in Sm B/B′ were also found to be dependent on PRMT7, including R108me1, R112me1, R132me1, R147me1, R172me1, and R181me1, in our dataset (FC > 2)^68^. Our RNA-seq analysis results led to the discovery that these three PRMTs hardly exhibited opposing effects on alternative splicing. In particular, a subset of alternative splicing events is commonly regulated by all three PRMTs. Considering that the final status of methyl-arginine was different for each PRMT, it was unexpected that they exhibited similar effects on alternative splicing.

Different statuses of arginine methylation have different effects on cellular function because of the different effector proteins they recognize^70–72^. For instance, asymmetrical and symmetrical dimethyl-arginine are associated with transcriptional activation and repression, respectively. Here, we demonstrated that regardless of its status, arginine methylation seemed to enhance RNA-protein interaction in general. We showed that arginine methylation enhanced the binding of hnRNPA1, a common substrate of PRMT4, 5, and 7, with pre-mRNA and, therefore, the alternative splicing events commonly regulated by all three PRMTs. The guanidino groups on the arginine side chain form interactions with biological hydrogen bond acceptors, which may be negatively altered by the addition of methyl groups. Alternatively, methylation can facilitate stacking with bases of RNA and DNA or aromatic residues^73^. As a consequence, arginine methylation has been shown to alter protein–protein and protein–RNA interactions^74^. The bulky methyl groups can prevent access to the potential hydrogen bond donors in arginine groups, thereby inhibiting protein interactions. Meanwhile, arginine methylation facilitates interaction with Tudor domains on proteins. A well-documented example is the arginine methylation of three of the seven Sm proteins (B/B′, D1, and D3), leading to their binding to the Tudor domain on SMN1^75^. Similarly, arginine methylation could either inhibit or facilitate protein–RNA interactions. For instance, the PRMT4-mediated methylation of the RNA binding protein p54nrb decreases its interaction with mRNA^76^. Meanwhile, Denman et al. have reported that methylation of the arginine-glycine-rich region in the fragile X mental retardation protein FMRP differentially affects RNA binding, and both increases and decreases of RNA binding are observed as a function of methylation^77^. Hsu et al. have shown that the PRMT3-mediated methylation of hnRNPA1 enhances its binding to *ABCG2* mRNA^78^. More recently, Wang et al. have demonstrated that the PRMT1-mediated methylation enhances HSP70 binding and *BCL2* mRNA stabilization via AU-rich elements in the 3′-UTR, which increased BCL2 protein expression and protected cancer cells from apoptosis induced by cellular stresses and therapeutics^79^.

Regarding the clinical significance of PRMT4, 5, and 7, as well as arginine methylation on hnRNPA1, they were highly expressed in several types of cancers, such as breast, colorectal, and prostate cancer, which is associated with aberrant alternative splicing events. The silencing or pharmacological inhibition of PRMT4, 5, and 7 was effective in suppressing cancer cell growth. The inhibition of PRMTs is a promising method for cancer therapy, which has been recently shown to be tightly linked to alternative splicing. Braun et al. have reported that PRMT5 knockdown or inhibition using GSK3235025 potently suppresses glioblastoma, which primarily disrupts the removal of detained introns (DIs) in proliferation genes. Thus, they revealed a PRMT5-regulated DI-splicing program as an exploitable cancer vulnerability^80^. Gerhart et al. have shown that PRMT5 inhibition using GSK3326595 activates the p53 pathway by inducing the alternative splicing of MDM4 in multiple cancer cell lines, which are critical determinants of the cellular response to PRMT5 inhibition. They suggested that the p53–MDM4 regulatory axis defines a subset of patients that could benefit from treatment with GSK3326595^81^. Fong et al. have revealed that the inhibition of PRMT5 and type I protein arginine methyltransferases (PRMTs) by GSK3203591 and MS023, respectively, reduces splicing fidelity and results in the preferential killing of SF (splicing factor)-mutant leukemias over wild-type counterparts and that their combination exhibits synergistic effects in killing tumor cells^55^. The methylation on many of arginine methylation sites identified in cultured cells was present in cancer cell samples, such as those on KRT1 and TAF15 proteins, further strengthening the functional importance of PRMT-mediated arginine methylation in cancers. Future studies should focus on the function of arginine methylation on these proteins to increased our understanding of how PRMT4, 5, and 7 contribute to cancer development.

Overall, our findings revealed the substrate specificity and commonality of type I, II, and III PRMTs, which were represented by PRMT4, 5, and 7, respectively, and increased our understanding of the arginine methylation network in cells. In particular, the PRMT4-, 5-, and 7-mediated methylation of splicing factors, such as hnRNPA1, plays non-redundant roles in RNA alternative splicing regulation, and such a mechanism was demonstrated to be potentially important for cancer development.

## Methods

### Cloning procedures

CCT7, TFG, YBX1, CKMT1B, hnRNPK, hnRNPA2B1, and △Np63α were PCR-amplified from cDNAs from HEK293 cells by using Transstart fastpfu fly polymerase (TransGen Biotech) and then cloned into pET-28a (Novagen) expression vector. hnRNPA1, PRMT4, 5, and 7 were similarly PCR-amplified and then cloned into pBoBi expression vector. 3 × Flag- and 3 × HA- tag were added to the amino- and carboxyl-terminus of hnRNPA1, PRMT4, 5, and 7, respectively. hnRNPA1 methylation deficient mutants (R/K (457), all the arginine residues methylated by PRMT4, 5 and, 7 were replaced by lysine; R/K (4), arginine residues methylated by PRMT4 were replaced by lysine; R/K (5), all the arginine residues methylated by PRMT5 were replaced by lysine; R/K (7), all the arginine residues methylated by PRMT7 were replaced by lysine) and PRMT4, 5, and 7 enzymatic dead mutants (PRMT4 (E257Q), PRMT5 (E435A, E444A), and PRMT7 (E144A, D147A, E153A)) were generated by overlap extension PCR method using Transstart fastpfu fly polymerase.

### Antibodies

Antibodies used in this study are listed as following: Rabbit monoclonal anti-mono-methyl arginine [mme-R] (Cell Signaling Technology, 8015S, 1:2000); PTMScan® mono-methyl arginine Motif [mme-RG] Kit (Cell Signaling Technology, 12235, for immunoenrichment); Rabbit monoclonal anti-symmetric di-methyl arginine motif [sdme-R] (Cell Signaling Technology, 13222S, 1:2000); PTMScan symmetric di-methyl arginine motif [sdme-RG] (Cell Signaling Technology, 13563, for immunoenrichment); Rabbit monoclonal anti-asymmetric di-methyl arginine motif [adme-RG] (Cell Signaling Technology, 13522S, 1:2000); PTMScan asymmetric di-methyl arginine motif [adme-RG] (Cell Signaling Technology, 13474, for immunoenrichment); mouse monoclonal anti-actin (Proteintech, 66009-1-Ig, 1:2000); Mouse monoclonal anti-CARM1(3H2) (Cell Signaling Technology, 12495S, 1:2000); Rabbit monoclonal anti-PRMT5 (EPR5772) (Abcam, ab109451, 1:2000); Rabbit monoclonal anti-PRMT7 (D1K6R) (Cell Signaling Technology, 14762S, 1:2000); Rabbit monoclonal anti-hnRNPA1 (Proteintech, 11176-1-AP, 1:2000); Rabbit monoclonal anti-hnRNPM (Proteintech, 26897-1-AP, 1:2000); Rabbit monoclonal anti-hnRNPA2B1 (Proteintech, 14813-1-AP, 1:2000); Rabbit monoclonal anti-hnRNPK (Proteintech, 11426-1-AP, 1:2000); Rabbit monoclonal anti-hnRNPU (Proteintech, 14599-1-AP, 1:2000); Mouse monoclonal anti-FLAG M2 (Sigma, F1804, 1:5000); Anti-FLAG M2 Affinity Gel (Sigma, A2220, for immunoenrichment); Monoclonal Anti-HA-Agarose, clone HA-7 (Sigma, A2095, for immunoenrichment).

### Clinical specimens

Breast tumor tissues and matched adjacent normal tissues were obtained from patients who were diagnosed with breast cancer and had undergone surgery at the Second Affiliated Hospital of Shantou University Medical College, which was approved by the Institutional Ethics Committee of the Second Affiliated Hospital of Shantou University Medical College. Colorectal tumor tissues and matched adjacent normal tissues were obtained from patients who were diagnosed with colorectal cancer and had undergone surgery at the First Affiliated Hospital of Xiamen University, which was approved by the Institutional Ethics Committee of the First Affiliated Hospital of Xiamen University. Prostate tumor tissues and matched adjacent normal tissues were obtained from prostatectomy surgery at Ren Ji Hospital, School of Medicine, Shanghai Jiao Tong University, which was approved by the Institutional Ethics Committee of the Ren Ji Hospital, School of Medicine, Shanghai Jiao Tong University. Tissue samples were freshly frozen in dry ice and stored at −80 °C until RNA extraction. All research was performed in compliance with government policies and the Helsinki Declaration. Experiments were undertaken with the understanding and written consent of each subject. The study is compliant with the “Guidance of the Ministry of Science and Technology (MOST) for the Review and Approval of Human Genetic Resources.”

### siRNA and plasmids transfection, and lenti-virus packaging and infection

Transfection of siRNA targeting PRMT4 (5′-GAUAGAAAUCCCAUUCAAA-3′ and 5′-GUAACCUCCUGGAUCUGAA-3′), PRMT5 (5′-GCACCAGTCTGTTCTGCTA-3′ and 5′-GGACCTGAGAGATGATATA-3′), PRMT7 (5′-GTCACAGAGTTGTTTGACA-3′ and 5′-GCAG ATATGCTACATGACA-3′), and hnRNPA1 (5′-CAGCTGAGGAAGCTCTTCA-3′) were performed using Lipofectamine 2000 (Invitrogen) according to the manufacturer’s protocol. Plasmid transfections in HEK293T cells were performed using PEI (Polyethyleneimine, Polysciences) according to the manufacturer’s protocol.

For Lenti-virus packaging: HEK293T cells were seeded in culture plates coated with poly-D-lysine (0.1% (w/v), Sigma, P7280) and transfected with lenti-viral vectors (pBoBi) together with packaging vectors, pMDL, VSVG, and REV, at a ratio of 10:5:3:2 using PEI for 48 h according to the manufacturer’s protocol. Virus were collected, filtered and added in the presence of 10 μg/mL polybrene (Sigma, H9268), followed by centrifugation for 30 min at 1500 × *g* at 37 °C. Medium was replaced 24 h later.

### Immunoblotting and immunoprecipitation

Cells were lysated in lysis buffer (50 mM Tris-HCl (pH 7.4), 150 mM NaCl, 1 mM EDTA and 1% Triton X-100) containing protease inhibitor cocktail (sigma, P2714-1BTL) on ice for 30 min followed by centrifugation. For immunoblotting, the resultant supernatant was directly boiled in SDS sample buffer, resolved by SDS-PAGE gel. For immunoprecipitation, the resultant supernatant was incubated with antibodies (2–5 μg) at 4 °C overnight. SureBeads Protein A or G magnetic beads (Bio-Rad, 161-4013 or 161-4023) were then added and incubated for an additional 4 hrs before washing five times with washing buffer (the same as lysis buffer). Beads were then re-suspended and boiled in SDS sample buffer, and the associated proteins were resolved by SDS-PAGE gel.

### Immunofluorescence

HEK293 cells were fixed with 4% paraformaldehyde in PBS for 20 min, and then permeabilized with 0.1% Triton X-100 in PBS on ice for 10 min. After rinsing with PBS buffer for three times, blocking solution (1% BSA in PBS) was applied for 1 h and primary antibody against Flag (Sigma, F1804, 1:200) was added in blocking buffer at 4 °C overnight. After washing with PBS/0.1% Triton X-100 for five times, cells were incubated with DAPI and secondary antibodies conjugated with Cy3 fluorescent dyes (Beyotime, a0521, 1:500) for 1 h, washed with PBS/0.1% Triton X-100, and mounted in Fluoromount-G (Southern Biotech). Images were recorded on a ZEISS Exciter 5 microscope (ZEISS).

### RNA isolation, RT-qPCR, and standard PCR

Total RNA was isolated using Trizol (Invitrogen) following the manufacturer’s protocol. First-strand cDNA synthesis from total RNA was carried out using GoScript™ Reverse Transcription Mix (Promega, random primers), followed by quantitative PCR (qPCR) using AriaMx Real-Time PCR machine (Agilent Technologies). RNA samples were subjected to qPCR to examine gene expression or standard PCR to examine alternative splicing change. Standard error of the mean is depicted. Sequence information for all primers used to check gene expression or alternative splicing was presented in Supplementary Data 9.

### RNA sequencing (RNA-seq), computational analysis of gene alternative splicing, and validation of gene alternative splicing

To prepare RNA for sequencing, total RNA was isolated using RNeasy Mini Kit (Qiagen) following the manufacturer’s protocol. DNase I in column digestion was included to ensure the RNA quality. RNA library preparation was performed by using NEBNext® UltraTM Directional RNA Library Prep Kit for Illumina(E7420L). Paired-end sequencing was performed with Illumina HiSeq platform at Amogene Biotech Co., Ltd.

BCL files were demultiplexed and converted to fastq files by using bcl2fastq (version 2.20), and then fastp (version 0.19.10) was used to trim adapter and filter out low quality reads. Sequencing reads were aligned to hg19 reference genome by using Tophat^82^ (http://ccb.jhu.edu/software/tophat/index.shtml). Cuffdiff was used to quantify the expression of RefSeq annotated genes with the option -M (reads aligned to repetitive regions were masked) and -u (multiple aligned reads are corrected using ‘rescue method’)^82^. Coding genes with FPKM (fragments per kilobase per million mapped reads) larger than 0.5 in any of the experimental conditions were included in our analysis. FPKM of a gene was calculated as mapped reads on exons divided by exonic length and the total number of mapped reads.

For splicing analysis, sequencing reads were aligned to hg19 reference genome by using Tophat. Alternative splicing events were detected by rMATS (version 3.2.5) with option “-novelSS 1” (detecting novel splice sites)^83^. Differential alternative splicing events were determined by PSI (| Δ PSI| = | PSI (siCTL) − PSI (siPRMT)| ≥ 0.5). PSI: percentage spliced in, long isoform divided by the sum of both short and long isoforms.

To search binding motifs for RNA-binding proteins, sequences from 25 to 600 bp upstream of alternative spliced exons were entered, and Ray2013 All Species (DNA-encoded) were selected to test for motif enrichment by CentriMo (http://meme-suite.org/tools/centrimo)^51^. CentriMo was designed for local motif enrichment analysis, which identifies known or user-provided motifs that show a significant preference for particular locations in the input sequences. CentriMO can also show if the local enrichment is significant relative to the control sequences.

Box plots were generated by R software and significance was determined using Student’s t-test. Heat maps were visualized using R software and pheatmap (V1.0.12).

Validation of alternative splicing events detected by RNA-seq was performed as reported previously^59^. Briefly, reverse transcription was carried out followed by standard PCR (RT-PCR) using primer sets specifically targeting the flanking exons of the alternatively spliced exon. The change of alternative splicing was presented as the change of PSI.

RNA-seq was deposited in the Gene Expression Omnibus database under accession GSE150040.

### Global mapping of PRMTs substrates

#### Cell labeling by SILAC (stable isotope labeling by amino acids in cell culture)

HEK293 cells were grown in SILAC DMEM (Invitrogen) supplemented with L-lysine/arginine (Sigma) (light label) or L-lysine/arginine-U-^13^C6 (Cambridge Isotope Laboratories) (heavy label) together with 10% dialyzed FBS, L-glutamine, and penicillin/streptomycin for 2 weeks (light labeled: arginine 84 mg/L and lysine 146 mg/L; heavy labeled: arginine 87.2 mg/L and lysine 152.8 mg/L). Cells were cultured at 37 °C in a humidified atmosphere containing 5% CO_2_. Light and heavy labeled cells were transfected with control siRNA and siRNA specifically targeting *PRMT4, 5 or 7*, respectively. Alternatively, light and heavy labeled cells were treated with or without SGC3027, respectively. Cells were then harvested, centrifuged (5 min, 500 × *g*), rinsed twice with ice-cold phosphate-buffered saline (PBS) and stored at −80 °C briefly before cell lysis.

#### Cell lysis and sample preparation

Cells were lysed in ten volumes of modified SDT buffer (0.1 M Tris-HCL, pH 7.6, 0.1 M DTT, 1% SDS, 1% SDC) and incubated at 95 °C for 5 min. The lysate was sonicated to shear genomic DNA, and clarified by centrifugation at 20,000 × *g* for 15 min at 20 °C. The supernatant was transferred to ultrafiltration units (Millipore, Amicon Ultra 15 Ultracel 10 KD) and centrifuged at 4000 × *g* for 40 min. After centrifugation, the concentrates were mixed with 2 mL of 50 mM iodoacetamide in UA solution (8 M urea, 100 mM Tris-HCl pH 8.5) and incubated in darkness at room temperature (RT) for 30 min followed by centrifugation for 30 min. After alkylation, the filter units were washed four times with 10 ml UA buffer and two times 10 mL of 50 mM ammonium bicarbonate by centrifugation at 4000 × *g*. Proteins were then digested with Lys-C (1:100, w/w, Wako) for 6 h at 37 °C and trypsin (1:50, w/w, Promega) overnight at 37 °C. The resulting peptide mixture was acidified (pH 2.0) with formic acid, loaded onto Sep-Pak tC18 cartridges (Waters), desalted and eluted with 70% acetonitrile. The eluted peptides were lyophilized and stored at −80 °C before analysis.

#### Offline high-pH fractionation

One hundred μg peptides (for proteome analysis) and 15 mg peptides (for methylome analysis) were off-line fractionated by bRP (basic Reverse Phase) using a Waters XBridge BEH C18 5 μm 4.6 × 250 mm column and XBridge BEH C18 10 μm 10 × 250 mm column on an Ultimate 3000 high-pressure liquid chromatography (HPLC) system (Dionex, Sunnyvale, CA, USA) operating at 1 mL/min and2.5 mL/min, respectively. Buffer A (5 mM ammonium formate) and buffer B (5 mM ammonium formate, 90% (v/v) ACN) were adjusted to pH 10 with ammonium hydroxide. Peptides were separated by a linear gradient from 5% B to 35% B in 54 min followed by a linear increase to 70% B in 6 min. A total of 60 fractions were collected. For comprehensive proteomic analysis, the 60 fractions were concatenated to 20. For comprehensive methylome analysis, the 60 fractions were concatenated to 10. All fractions were monitored at 214 nm UV wave length. All the concentrated fractions were lyophilized. Peptides were quantified by nanodrop and 1 μg of peptides were injected into the column for each run.

#### Enrichment of mono-, asymmetrical di- and symmetrical di-methylated arginine peptides

The lyophilized peptides were dissolved by 600 μl 1 × PTMScan IAP Buffer (CST). PTMScan mono-methyl arginine motif immunoaffinity beads (CST) were incubated with the 10 dissolved fractions for 4 h at 4 °C. The PTMScan® mono-methyl arginine motif [mme-RG] Kit #12235 for immunoenrichment we used is a mixture of two antibodies, mono-methyl arginine [mme-R] MultiMab™ Rabbit mAb mix (CST, Cat# 8015, used to be called Me-R4-100 antibody) and mono-methyl arginine (R*GG) (D5A12) Rabbit mAb (CST, Cat# 8711). Mono-methyl arginine [mme-R] MultiMab™ Rabbit mAb mix was generated by using a library of synthetic monomethylarginine peptides (XXXXXXXR*XXXXXX) as antigen, which can specifically recognize monomethylarginine and has no sequence preference. In contrast, a library of synthetic monomethylarginine peptides containing the R*GG motif (XXXXXXXR*GGXXX) was used to immunize animals to produce mono-methyl arginine (R*GG) (D5A12) Rabbit mAb. This antibody also specifically recognizes monomethylarginine. After enrichment with mono-methylated antibodies, the supernatant from the 10 fractions were concatenated to 5. PTMScan asymmetric di- or symmetrical di-methyl arginine motif immunoaffinity beads (CST, 13563, and 13474) were incubated with the 5 fractions for 4 h at 4 °C. All the immunoprecipitates were washed three times in ice-cold immunoprecipitation buffer followed by three washes in water, and modified peptides were eluted with 2 × 50 μl of 0.15% trifluoroacetic acid in Milli-Q water. Peptide eluates were desalted on reversed-phase C18 StageTips and dried by speed vacuum at 45 °C.

#### LC-MS/MS analysis

All MS experiments were performed on a nanoscale EASY-nLC 1200UHPLC system (Thermo Fisher Scientific) connected to an Orbitrap Fusion Lumos equipped with a nanoelectrospray source (Thermo Fisher Scientific). Mobile phase A contained 0.1% formic acid (v/v) in water; mobile phase B contained 0.1% formic acid in 80% acetonitrile (ACN). The peptides were dissolved in 0.1% formic acid (FA) with 2% acetonitrile and separated on a RP-HPLC analytical column (75 μm × 25 cm) packed with 2 μm C18 beads (Thermo Fisher Scientific) using a linear gradient ranging from 8% to 30% ACN in 90 min and followed by a linear increase to 44% B in 20 min at a flow rate of 300 nL/min. The Orbitrap Fusion Lumos acquired data in a data-dependent manner alternating between full-scan MS and MS2 scans. The spray voltage was set at 2.2 kV and the temperature of ion transfer capillary was 300 °C. The MS spectra (350–1500 *m/z*) were collected with 120,000 resolutions, AGC of 4 × 10^5^, and 50 ms maximal injection time. Selected ions were sequentially fragmented in a 3 seconds (s) cycle by HCD with 30% normalized collision energy, specified isolated windows 1.6 *m/z*, and 30,000 resolutions. AGC of 5 × 10^4^ and 150 ms maximal injection time were used. Dynamic exclusion was set to 15 s. Unassigned ions or those with a charge of 1+ and >7+ were rejected for MS/MS. MS and MS/MS data were acquired using the Xcalibur software (Thermo Fisher Scientific, v4.0).

#### Mass spectrometry data analysis

Raw data were processed using Proteome Discoverer (PD, Thermo Fisher Scientific, v2.2), and MS/MS spectra were searched against the SwissProt human database (downloaded in April 2018), and the total number of entries is 20,259. All searches were carried out with precursor mass tolerance of 20 ppm, fragment mass tolerance of 0.02 Da, oxidation (Met) (+15.9949 Da) and acetylation (protein N-terminus) (+42.0106 Da) as variable modifications, carbamidomethylation (Cys) (+57.0215 Da) as fixed modification, and three trypsin missed cleavages allowed when searching for proteins. All searches were carried out with precursor mass tolerance of 20 ppm, fragment mass tolerance of 0.02 Da, oxidation (Met) (+15.9949 Da), methylation (Arg, Lys) (+14.0266 Da), dimethylation (Arg, Lys) (+28.0532 Da), trimethylation (Lys) (+42.0470 Da) and acetylation (protein N-terminus) (+42.0106 Da) as variable modifications, carbamidomethylation (Cys) (+57.0215 Da) as fixed modification and three trypsin missed cleavages allowed when searching for methylation. Only peptides with at least six amino acids in length were considered. The peptide and protein identifications were filtered by PD to control the false discovery rate (FDR) < 1%. At least one unique peptide was required for protein identification.

#### Bioinformatics analysis

Mono- and di-methylation sites were first extracted. Motif analysis was performed by using the IceLogo web server taking 5 amino acids (AAs) upstream and downstream of the arginine methylation site identified (11 AAs in total)^84^. Mutation frequency at or in the vicinity of PRMTs-dependent arginine sites (5 AAs upstream and downstream) was calculated based on the COSMIC database^45^. Similar analysis was performed for any arginine site in the human proteome, and P value was calculated by the Fisher’s exact test. Gene ontology (GO) and KEGG (Kyoto Encyclopedia of Genes and Genomes) pathway analysis on PRMTs substrates was performed using the Metascape web server^46^.

All mass spectrometry raw files have been deposited in ProteomeXchange via the PRIDE database with the accession number PXD022424.

### TMT (tandem mass tags) quantitative proteomics

#### Sample preparation

The tissue samples were processed according to the Filter Aided Sample Preparation (FASP) method^85^. Briefly, tissue samples were lysed in ten volumes of modified SDT buffer (0.1 M Tris. HCl, pH 7.6, 0.1 M DTT, 3% SDS, 1% SDC) and incubated for 5 min at 95 °C. The lysates were then sonicated to shear genomic DNA and clarified by centrifugation at 20,000 × *g* for 15 min at 20 °C. The resultant supernatant was transferred to Amicon Ultra 4 Ultracel 10 KD ultrafiltration units (Millipore) and centrifuge for 40 min at 4000 × *g*. After centrifugation, the concentrates were mixed with 1 mL of 50 mM iodoacetamide in UA solution (8 M urea, 100 mM Tris. HCl, pH 8.5) and incubated in darkness at room temperature (RT) for 30 min followed by centrifugation for 30 min. After alkylation, the filter units were washed four times with 4 mL of UA buffer and 10 mL of 50 mM ammonium bicarbonate for two times by centrifugation at 4000 × *g*. Proteins were then digested with Lys-C (1:100 (w/w), wako) for 6 h at 37 °C and trypsin (1:50 (w/w), Promega) overnight at 37 °C. The resulting peptide mixture was acidified with formic acid (pH, 2). The tryptic peptides were desalted by StageTips and lyophilized followed by labeling with TMT10plex (Pierce) according to the manufacturer’s instructions. Two hundred μg labeled peptides were off-line fractionated by bRP using a Waters XBridge BEH C18 5 μm 4.6 × 250 mm column (Waters) on an Ultimate 3000 high-pressure liquid chromatography (HPLC) system (Dionex) operating at 1 mL/min. Buffer A (5 mM ammonium formate) and buffer B (5 mM ammonium formate, 90% (v/v) ACN) were adjusted to pH 10 with ammonium hydroxide. Peptides were separated by a linear gradient from 5% B to 40% B in 90 min followed by a linear increase to 70% B in 6 min. A total of 96 fractions were collected. The 96 fractions were concatenated to 32 fractions, and all the peptide fractions were lyophilized. Peptides were quantified by nanodrop and 1 μg of peptides were injected into the column for each run.

#### LC-MS/MS analysis

MS experiments were performed on a nanoscaleEASY-nLC 1200UHPLC system (Thermo Fisher Scientific) connected to an Orbitrap Fusion Lumos equipped with a nanoelectrospray source (Thermo Fisher Scientific). Mobile phase A contained 0.1% formic acid (v/v) in water; mobile phase B contained 0.1% formic acid in 80% acetonitrile (ACN). The peptides were dissolved in 0.1% formic acid (FA) with 2% ACN and separated on a RP-HPLC analytical column (75 μm × 25 cm) packed with 2 μm C18 beads (Thermo Fisher Scientific) using a linear gradient ranging from 9 to 32% ACN in 100 min and followed by a linear increase to 50% B in 20 min at a flow rate of 300 nL/min. The Orbitrap Fusion Lumos acquired data in a data-dependent manner alternating between full-scan MS and MS2 scans. The spray voltage was set at 2.2 kV and the temperature of ion transfer capillary was 300 °C. The MS spectra (350–1500 *m/z*) were collected with 60,000 resolution, AGC of 4 × 10^5^ and 50 ms maximal injection time. Selected ions were sequentially fragmented in a 3 seconds (s) cycle by HCD with 38% normalized collision energy, specified isolated windows 0.7 *m/z* and 50,000 resolution. AGC of 1 × 10^5^ and 105 ms maximal injection time were used. Dynamic exclusion was set to 30 s. Unassigned ions or those with a charge of 1+ and >7+ were rejected for MS/MS. MS and MS/MS data were acquired using the Xcalibur software (Thermo Fisher Scientific, version 4.0).

#### Mass spectrometry data analysis

Raw data were processed using Proteome Discoverer (PD, Thermo Fisher Scientific, version 2.2), and MS/MS spectra were searched against the SwissProt human database (downloaded in April 2018), and the total number of entries is 20,259. All searches were carried out with precursor mass tolerance of 20 ppm, fragment mass tolerance of 0.02 Da, oxidation (Met) (+15.9949 Da), TMT6plex (Lys) (229.163 Da) and acetylation (protein N-terminus) (+42.0106 Da) as variable modifications, carbamidomethylation (Cys) (+57.0215 Da), TMT6plex (N-terminal) (229.163 Da) as fixed modification and three trypsin missed cleavages allowed when searching for proteins. All searches were carried out with precursor mass tolerance of 20 ppm, fragment mass tolerance of 0.02 Da, oxidation (Met) (+15.9949 Da), TMT6plex(Lys)(229.163 Da), methylation (Arg, Lys) (+14.0266 Da), di-methylation (Arg, Lys) (+28.0532 Da), tri-methylation (Lys) (+42.0470 Da) and acetylation (protein N-terminus) (+42.0106 Da) as variable modifications, carbamidomethylation (Cys) (+57.0215 Da), TMT6plex (N-terminal) (229.163 Da) as fixed modification and three trypsin missed cleavages allowed when searching for methylation. Only peptides with at least six amino acids in length were considered. The peptide and protein identifications were filtered by PD to control the false discovery rate (FDR) < 1%. At least one unique peptide was required for protein identification.

### MALDI-TOF MS analysis

In vitro methylation reactions were first desalted with ZipTip (C18, Millipore) according to the manufacturer’s protocol with minor modifications. Briefly, ZipTip was pre-washed with 100% ACN, and then equilibrated with 0.1% formic acid (FA) for 10 times. Samples were adjusted to pH 2–3 using FA, and then loaded on ZipTip by slowly aspirating and dispensing for 10 times, followed by washing with 0.1% FA for 10 times. The peptides were finally eluted using 70% ACN with 0.1% FA. 2,5-Dihydroxybenzoic acid (Sigma) matrix was prepared at a concentration of 20 mg/mL in ACN/water (1:1, v/v) with 0.1% FA. Samples were prepared by applying 1 μl mixture solution (1:1, v/v) of sample and matrix onto the stainless steel MALDI target plate, allowing the droplet to dry in the air at room temperature (RT) before transferring into mass spectrometer. MALDI-TOF MS analyses were performed on a Bruker Autoflex II mass spectrometer (Bruker Daltonics) in a positive reflection mode. The mass spectrometer was equipped with a pulsed nitrogen laser operated at 337 nm with 3 ns duration pulses and employed stainless steel targets (MTP 384 target ground steel, Bruker Daltonics). Voltage impressed on the ion one and two was 20.0 and 19.0 kV, respectively. The laser power energy was adjusted as needed. The acceleration voltage, grid voltage and delayed extraction time were set as 19.0 kV, 90% and 150 ns, respectively.

### Cell proliferation assay

Cell viability was measured by using a CellTiter 96 AQueous one solution cell proliferation assay kit (Promega) following the manufacturer’s protocol. Briefly, cells were either transfected with siRNAs or treated with EZM2302 (ProbeChem), GSK591 (TOPSCIENCE), and SGC3027 (MCE) and then maintained in culture medium for different time points followed by cell proliferation assay. To measure cell viability, 20 μl of CellTiter 96 AQueous one solution reagent was added per 100 μl of culture medium, and the culture plates were incubated for 1 h at 37 °C in a humidified, 5% CO_2_ atmosphere incubator. The reaction was stopped by adding 25 μl of 10% SDS. Data was recorded at wavelength 490 nm using a Thermo Multiskan MK3 Microplate Reader.

### Protein purification and in vitro methylation assay

His-tagged CCT7, TFG, YBX1, CKMT1B, hnRNPK, hnRNPA2B1, and ΔNp63α were expressed in BL21 (DE3) bacterial cells (Stratagene) and purified by using Ni-NTA Resin (Thermofisher).

HA-tagged PRMT7 was expressed in HEK293T and lysed in a lysis buffer containing 50 mM Tris-HCl (pH 7.4), 420 mM NaCl, 1 mM EDTA, and 1% Triton X-100 supplemented with complete protease inhibitor cocktail followed by affinity purification by using anti-Flag M2 agarose and washed extensively with washing buffer containing 50 mM Tris-HCl, pH 7.4, 420 mM NaCl, 1 mM EDTA, 1% Triton X-100 before elution with 3 × Flag peptides (Sigma).

In vitro methylation assay was performed in methylation buffer (50 mM Tris-HCl, pH 8.0, 20 mM KCl, 5 mM DTT, 4 mM EDTA) in the presence of methionine at 37 °C for 1 h. The reaction was stopped by adding SDS sample buffer followed by SDS-PAGE gel and immunoblotting.

### RNA Immunoprecipitation (RNA-IP)

Cells over-expressing 3 × Flag-tagged wild-type or mutant hnRNPA1 were lysed in polysome lysis buffer (100 mM KCl, 5 mM MgCl2, 10 mM HEPES (pH 7.0), 0.5% NP-40, 1 mM DTT, 100 U/ml RNasin RNase inhibitor (Promega, N2511), 2 mM vanadyl ribonucleoside complexes solution (Sigma, 94742), 25 μl/ml protease inhibitor cocktail (Sigma, P8340)), which were then subjected to IP by using M2 agarose (Sigma, F1804) followed by washing with polysome lysis buffer four times and polysome lysis buffer plus 1 M urea four times. RNAs was released by adding 150 μl of polysome lysis buffer with 0.1% SDS and 45 μg proteinase K (Ambion, AM2548) and incubated at 50 °C for 30 min. RNA extracted with phenol-chloroform-isoamyl alcohol mixture (Sigma, 77618) was recovered by adding 2 μl GlycoBlue (15 mg/ml, Ambion, AM9516), 36 μl 3 M sodium acetate and 750 μl ethanol followed by incubation at −20 °C for overnight. Precipitated RNAs were washed with 70% ethanol, air dried, and re-suspended in RNase free water followed by DNase I (Promega, M6101) treatment to remove genomic DNA. The resultant RNAs were subjected to RT-qPCR analysis.

### Reporting summary

Further information on research design is available in the Nature Research Reporting Summary linked to this article.

## Supplementary information

Supplementary information

Description of Additional Supplementary Files

Supplementary Data 1

Supplementary Data 2

Supplementary Data 3

Supplementary Data 4

Supplementary Data 5

Supplementary Data 6

Supplementary Data 7

Supplementary Data 8

Supplementary Data 9

Supplementary Data 10

Supplementary Data 11

Reporting Summary

## Data Availability

All mass spectrometry raw files have been deposited in ProteomeXchange via the PRIDE database with the accession number PXD022424. RNA-seq was deposited in the Gene Expression Omnibus database under accession GSE150040. All other data are available from the corresponding authors upon request. Source data are provided with this paper.

## Source data

Source data
