# Supplementary material for: Profiling PRMT methylome reveals roles of hnRNPA1 arginine methylation in RNA splicing and cell growth
Source: Nat Commun. 2021 Mar 29;12:1946. doi: 10.1038/s41467-021-21963-1 (PMC8007824; doi:10.1038/s41467-021-21963-1)
Supplement: Supplementary file 1 — Supplementary information [file 41467_2021_21963_MOESM1_ESM.pdf]

a

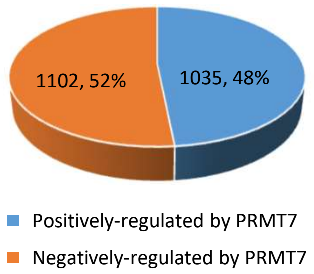

b

All Rme1 sites	Rme1 sites that could be quantified	Rme1 sites with at least 2 fold reduction of methylation signals in SGC3027-treated cells	Rme1 sites with abolished methylation signals in SGC3027-treated cells
1126 (Rme1)	1027 (Rme1)	503 (Rme1)	337 (Rme1)
524 (protein)	474 (protein)	274 (protein)	194 (protein)

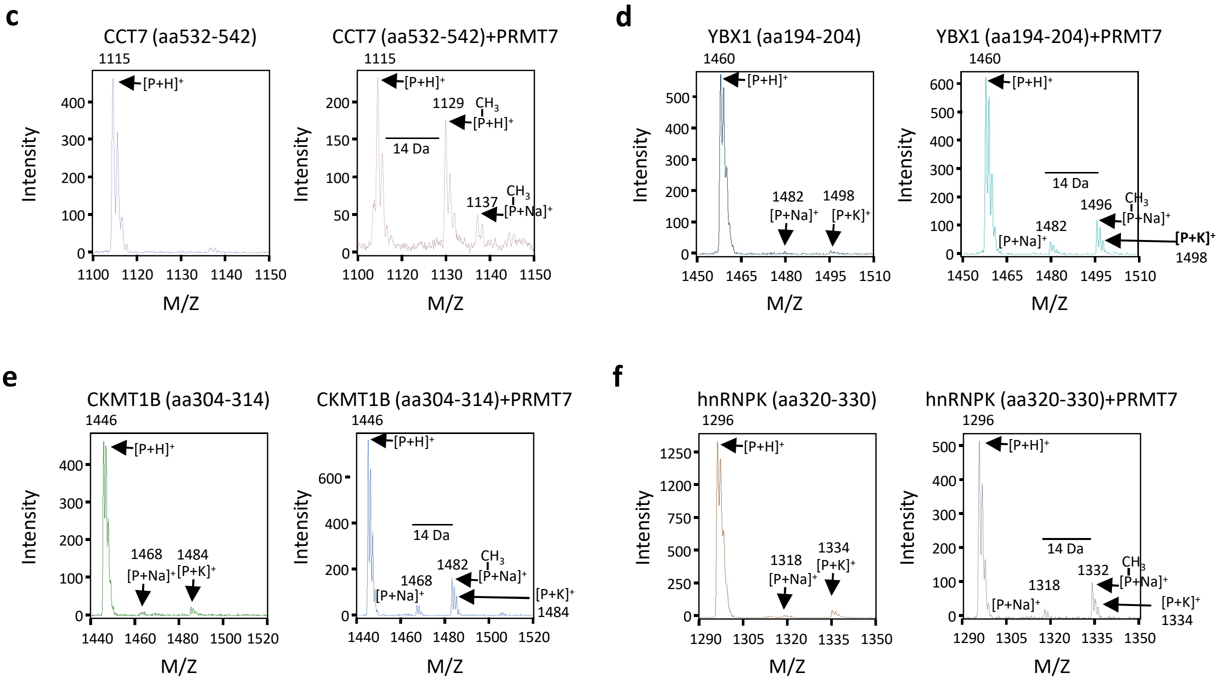

Supplementary figure 1. PRMT7 knockdown changed the expression of a large number of proteins. (a) SILAC labelled wild-type (WT) (light) and PRMT7-knockdown (heavy) HEK293 cells was subjected to quantitative proteomics analysis as shown in Fig. 1d. Protein regulated by PRMT7 is shown (FC $\geq$ 2). (b) The number of mono-methyl arginine (Rme1) sites detected (column 1), Rme1 sites could be quantified (column 2), Rme1 sites with methylation signals decreased at least two-fold (column 3) or abolished (column 4) in mass spectrometry analysis when cells were treated with PRMT7 inhibitor SGC3027 as described in Fig. 1d was shown. The number of proteins encompass all these methylation sites was also shown (bottom lane). (c-f) *In vitro* methylation assay was performed by mixing purified PRMT7 with synthetic short peptides from CCT7 (c), YBX1 (d), CKMT1B (e), and hnRNPK (f) proteins. The reactions were then subjected to MALDI-TOF MS analysis. P, peptide; H, proton; Na, sodium; K, potassium; CH3, methyl group; aa, amino acid; M/Z, mass to charge (M/Z).

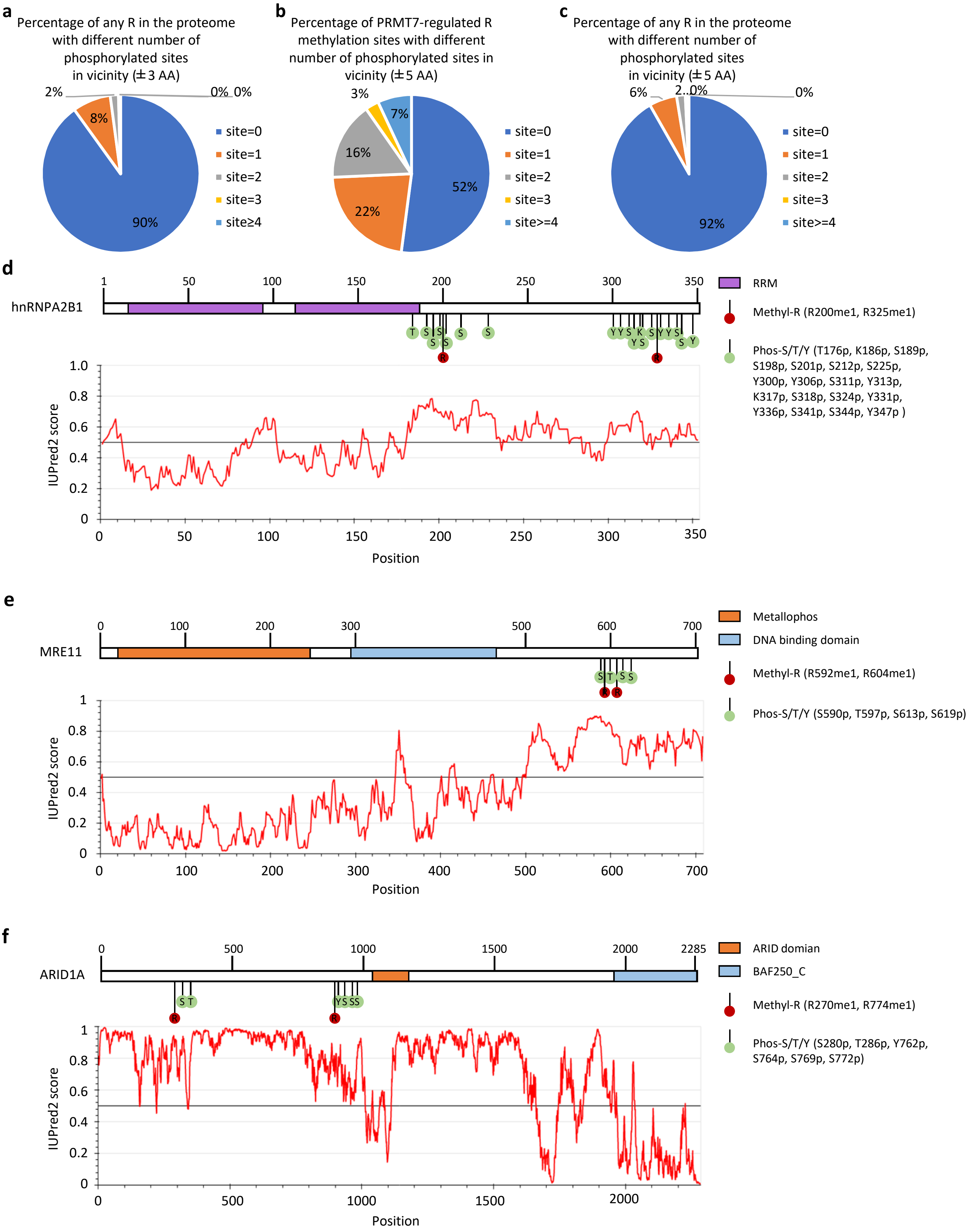
a
Percentage of any R in the proteome with different number of phosphorylated sites in vicinity (±3 AA)
2%
0% 0%
8%
90%
site=0
site=1
site=2
site=3
site≥4
b
Percentage of PRMT7-regulated R methylation sites with different number of phosphorylated sites in vicinity (±5 AA)
3%
7%
16%
52%
22%
site=0
site=1
site=2
site=3
site>=4
c
Percentage of any R in the proteome with different number of phosphorylated sites in vicinity (±5 AA)
6%
2..0%
0%
92%
site=0
site=1
site=2
site=3
site>=4
d
hnRNPA2B1
RRM
Methyl-R (R200me1, R325me1)
Phos-S/T/Y (T176p, K186p, S189p, S198p, S201p, S212p, S225p, Y300p, Y306p, S311p, Y313p, K317p, S318p, S324p, Y331p, Y336p, S341p, S344p, Y347p)
IUPred2 score
Position
e
MRE11
Metallophos
DNA binding domain
Methyl-R (R592me1, R604me1)
Phos-S/T/Y (S590p, T597p, S613p, S619p)
IUPred2 score
Position
f
ARID1A
ARID domian
BAF250_C
Methyl-R (R270me1, R774me1)
Phos-S/T/Y (S280p, T286p, Y762p, S764p, S769p, S772p)
IUPred2 score
Position

Supplementary figure 2. Co-occurrence of phosphorylation and PRMT7-mediated arginine methylation. (a) The percentage of any arginine (R) site in the proteome with different number of phosphorylation sites (n = 0, 1, 2, 3 or ≥ 4) in vicinity (± 3 amino acid (AA) window) is shown. (b, c) The percentage of PRMT7-regulaed arginine methylation sites (b) or any arginine sites (c) in the proteome with different number of phosphorylation sites (n = 0, 1, 2, 3 or ≥ 4) in vicinity (± 5 amino acid (AA) window) is shown. (d-f) Schematic representation of the domain architecture of representative proteins with co-occurrence of phosphorylation and PRMT7-regulated arginine methylation sites (upper panel). The disorder tendency was predicted by using IUPred12 (http://iupred.enzim.hu) (bottom panel). S: serine; T: threonine; Y: tyrosine; R: arginine; p: phosphorylation; me: methylation.

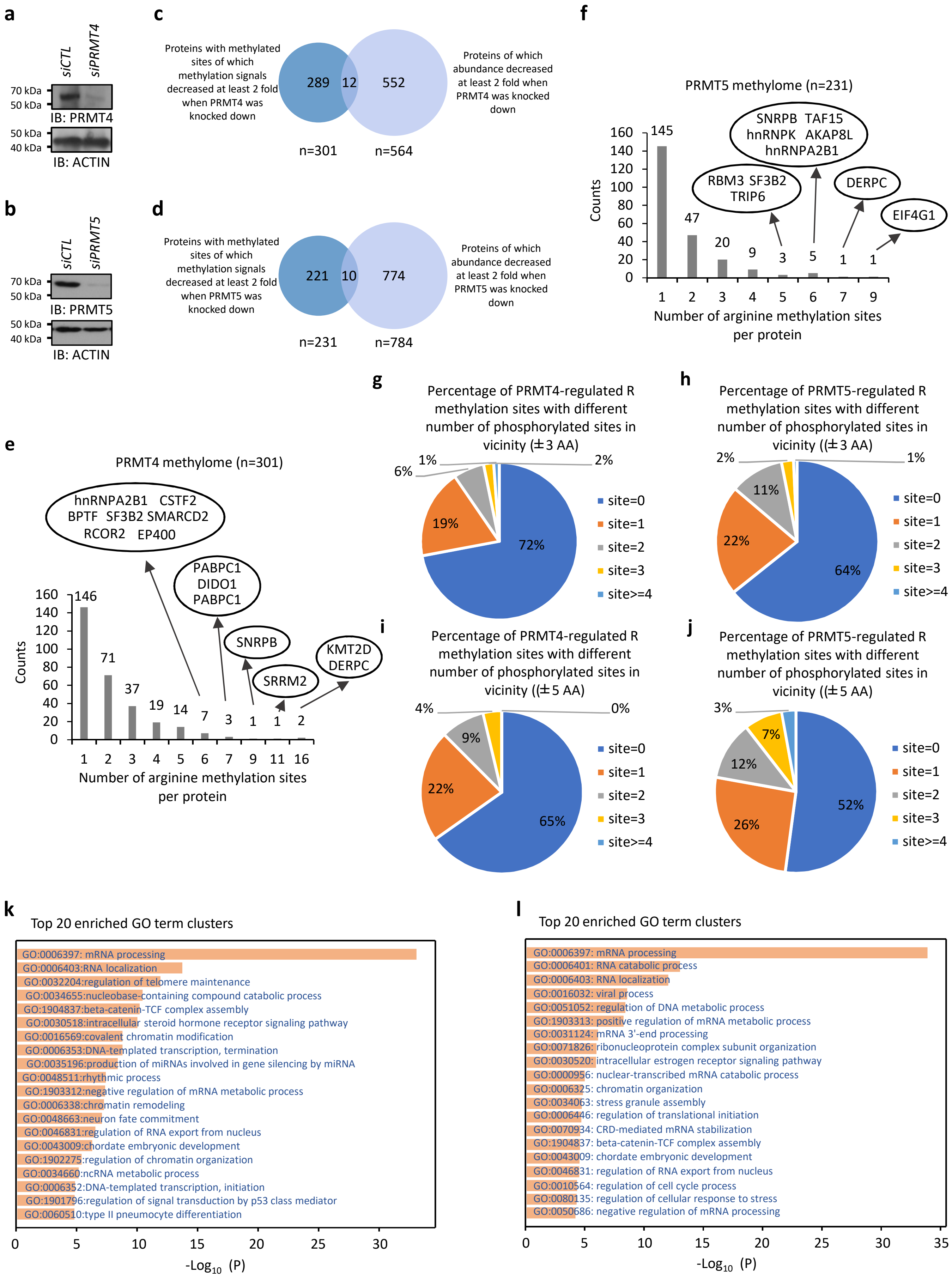

a
siCTL siPRMT4
70 kDa
50 kDa
IB: PRMT4
50 kDa
40 kDa
IB: ACTIN
b
siCTL siPRMT5
70 kDa
50 kDa
IB: PRMT5
50 kDa
40 kDa
IB: ACTIN
c
Proteins with methylated sites of which methylation signals decreased at least 2 fold when PRMT4 was knocked down
289 12 552
Proteins of which abundance decreased at least 2 fold when PRMT4 was knocked down
n=301 n=564
d
Proteins with methylated sites of which methylation signals decreased at least 2 fold when PRMT5 was knocked down
221 10 774
Proteins of which abundance decreased at least 2 fold when PRMT5 was knocked down
n=231 n=784
e
PRMT4 methylome (n=301)
hnRNPA2B1 CSTF2 BPTF SF3B2 SMARCD2 RCOR2 EP400
PABPC1 DIDO1 PABPC1
SNRPB
SRRM2
KMT2D DERPC
Counts
Number of arginine methylation sites per protein
f
PRMT5 methylome (n=231)
SNRPB TAF15 hnRNPK AKAP8L hnRNPA2B1
RBM3 SF3B2 TRIP6
DERPC
EIF4G1
Counts
Number of arginine methylation sites per protein
g
Percentage of PRMT4-regulated R methylation sites with different number of phosphorylated sites in vicinity (±3 AA)
h
Percentage of PRMT5-regulated R methylation sites with different number of phosphorylated sites in vicinity ((±3 AA)
i
Percentage of PRMT4-regulated R methylation sites with different number of phosphorylated sites in vicinity ((±5 AA)
j
Percentage of PRMT5-regulated R methylation sites with different number of phosphorylated sites in vicinity ((±5 AA)
site=0
site=1
site=2
site=3
site>=4
k
Top 20 enriched GO term clusters
GO:0006397: mRNA processing
GO:0006403:RNA localization
GO:0032204:regulation of telomere maintenance
GO:0034655:nucleobase-containing compound catabolic process
GO:1904837:beta-catenin-TCF complex assembly
GO:0030518:intracellular steroid hormone receptor signaling pathway
GO:0016569:covalent chromatin modification
GO:0006353:DNA-templated transcription, termination
GO:0035196:production of miRNAs involved in gene silencing by miRNA
GO:0048511:rhythmic process
GO:1903312:negative regulation of mRNA metabolic process
GO:0006338:chromatin remodeling
GO:0048663:neuron fate commitment
GO:0046831:regulation of RNA export from nucleus
GO:0043009:chordate embryonic development
GO:1902275:regulation of chromatin organization
GO:0034660:ncRNA metabolic process
GO:0006352:DNA-templated transcription, initiation
GO:1901796:regulation of signal transduction by p53 class mediator
GO:0060510:type II pneumocyte differentiation
-Log10 (P)
l
Top 20 enriched GO term clusters
GO:0006397: mRNA processing
GO:0006401: RNA catabolic process
GO:0006403: RNA localization
GO:0016032: viral process
GO:0051052: regulation of DNA metabolic process
GO:1903313: positive regulation of mRNA metabolic process
GO:0031124: mRNA 3'-end processing
GO:0071826: ribonucleoprotein complex subunit organization
GO:0030520: intracellular estrogen receptor signaling pathway
GO:0000956: nuclear-transcribed mRNA catabolic process
GO:0006325: chromatin organization
GO:0034063: stress granule assembly
GO:0006446: regulation of translational initiation
GO:0070934: CRD-mediated mRNA stabilization
GO:1904837: beta-catenin-TCF complex assembly
GO:0043009: chordate embryonic development
GO:0046831: regulation of RNA export from nucleus
GO:0010564: regulation of cell cycle process
GO:0080135: regulation of cellular response to stress
GO:0050686: negative regulation of mRNA processing
-Log10 (P)

Supplementary figure 3. Comparison among PRMT7-, 4-, and 5-regulated arginine methylation revealed the common features of these three PRMTs. (a, b) HEK293 cells transfected with *siCTL* or *siPRMT4* or *siPRMT5* were analyzed by immunoblotting with anti-PRMT4 (a) or PRMT5 (b) antibodies. ACTIN was served as a loading control. (c) Overlap between proteins in PRMT4 methylome and proteins which abundance was decreased at least two-fold when PRMT4 was knocked down is shown. (d) Overlap between proteins in PRMT5 methylome and proteins which abundance was decreased at least two-fold when PRMT5 was knocked down is shown. (e, f) The distribution of proteins in PRMT4 (e) or PRMT5 (f) methylome with different number of arginine methylation sites. The proteins with more than two methylation sites are shown in oval. (g-j) The percentage of PRMT4- (g, i) and PRMT5- (h, j) regulated arginine methylation sites with different number of phosphorylation sites (n = 0, 1, 2, 3, or $\geq$ 4) in vicinity ($\pm$ 3 (g, h) or $\pm$ 5 (i, j) amino acid (AA) window) is shown. (k, l) Gene ontology (GO) analysis using Metascape[46] for PRMT4 (k) and PRMT5 (l) methylome. Representative terms from the top 20 enriched GO term clusters are shown. Source data are provided as a Source Data file.

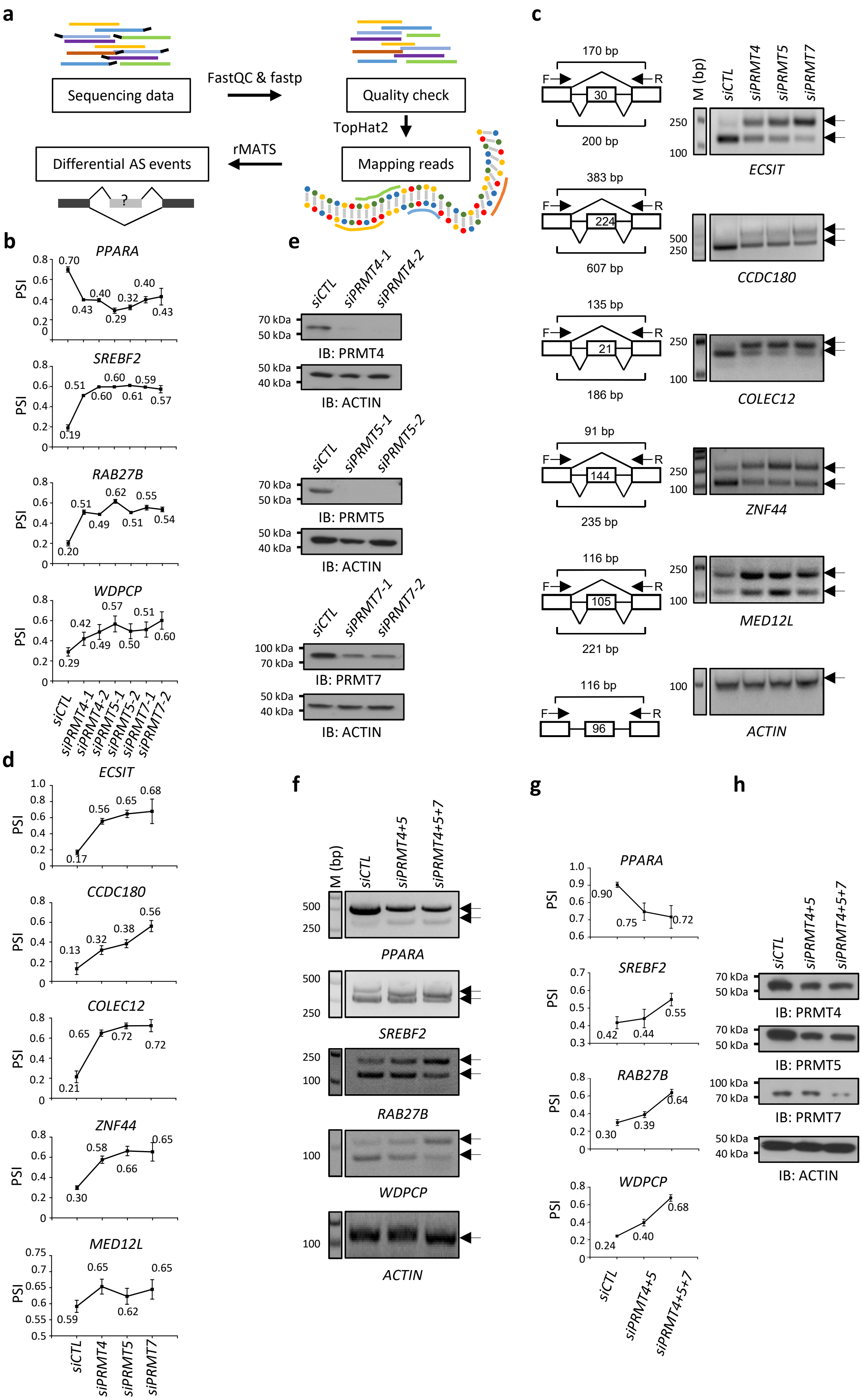
a
Sequencing data
FastQC & fastp
Quality check
TopHat2
Mapping reads
rMATS
Differential AS events
b
PPARA
SREBF2
RAB27B
WDPCP
PSI
siCTL
siPRMT4-1
siPRMT4-2
siPRMT5-1
siPRMT5-2
siPRMT7-1
siPRMT7-2
c
170 bp
200 bp
383 bp
607 bp
135 bp
186 bp
91 bp
235 bp
116 bp
221 bp
M (bp)
ECSIT
CCDC180
COLEC12
ZNF44
MED12L
ACTIN
d
e
IB: PRMT4
IB: ACTIN
IB: PRMT5
IB: PRMT7
f
siPRMT4+5
siPRMT4+5+7
g
h

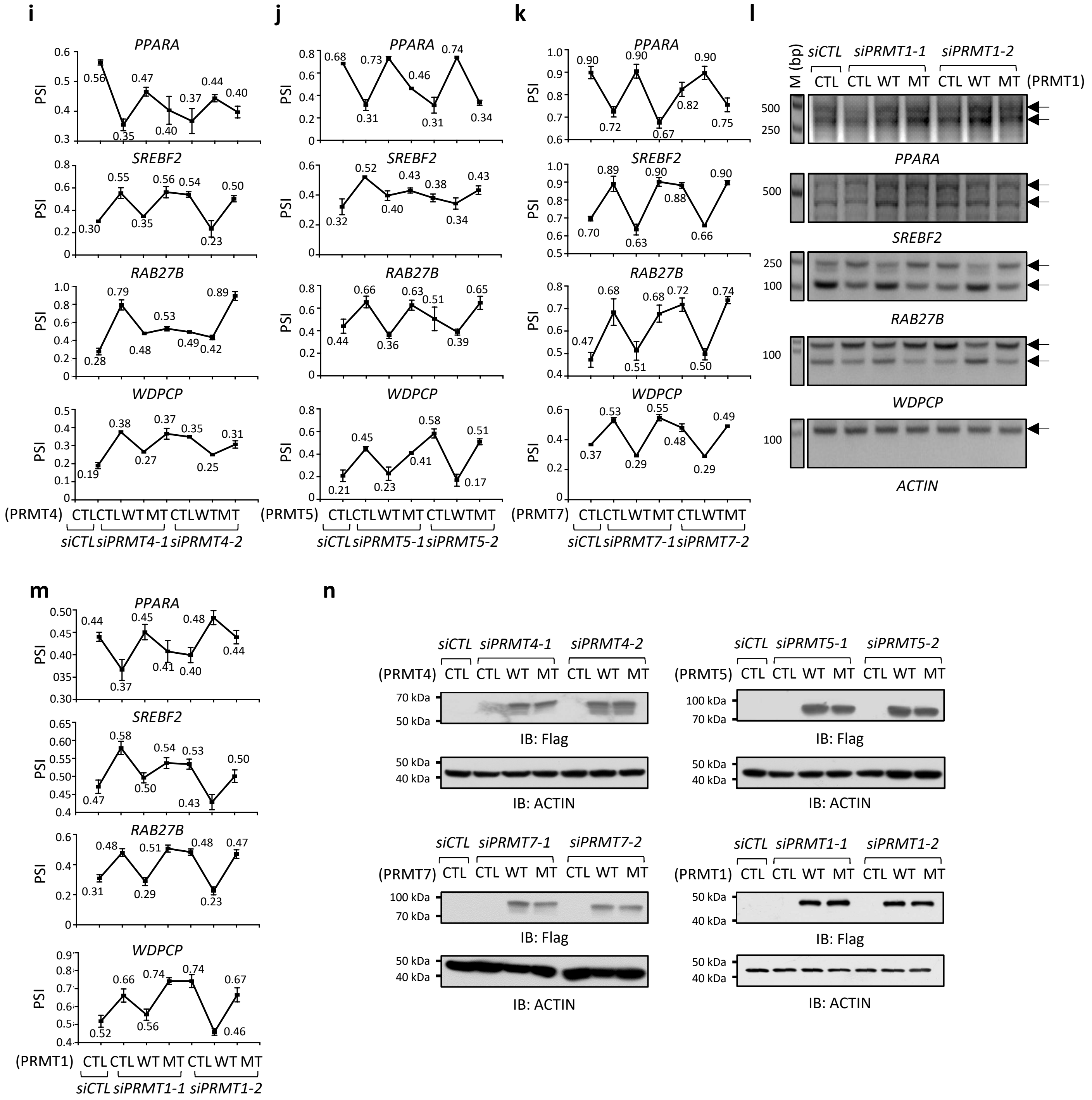

i
PPARA
SREBF2
RAB27B
WDPCP
PSI
(PRMT4) CTL CTL WT MT CTL WT MT
siCTL siPRMT4-1 siPRMT4-2
j
PPARA
SREBF2
RAB27B
WDPCP
PSI
(PRMT5) CTL CTL WT MT CTL WT MT
siCTL siPRMT5-1 siPRMT5-2
k
PPARA
SREBF2
RAB27B
WDPCP
PSI
(PRMT7) CTL CTL WT MT CTL WT MT
siCTL siPRMT7-1 siPRMT7-2
l
M (bp)
siCTL siPRMT1-1 siPRMT1-2
CTL CTL WT MT CTL WT MT (PRMT1)
500
250
PPARA
500
SREBF2
250
100
RAB27B
100
WDPCP
100
ACTIN
m
PPARA
SREBF2
RAB27B
WDPCP
PSI
(PRMT1) CTL CTL WT MT CTL WT MT
siCTL siPRMT1-1 siPRMT1-2
n
siCTL siPRMT4-1 siPRMT4-2
(PRMT4) CTL CTL WT MT CTL WT MT
70 kDa
50 kDa
IB: Flag
50 kDa
40 kDa
IB: ACTIN
siCTL siPRMT5-1 siPRMT5-2
(PRMT5) CTL CTL WT MT CTL WT MT
100 kDa
70 kDa
IB: Flag
50 kDa
40 kDa
IB: ACTIN
siCTL siPRMT7-1 siPRMT7-2
(PRMT7) CTL CTL WT MT CTL WT MT
100 kDa
70 kDa
IB: Flag
50 kDa
40 kDa
IB: ACTIN
siCTL siPRMT1-1 siPRMT1-2
(PRMT1) CTL CTL WT MT CTL WT MT
50 kDa
40 kDa
IB: Flag
50 kDa
40 kDa
IB: ACTIN

Supplementary figure 4. RNA-seq profiling results revealed that PRMT4, 5, and 7 exhibited a global impact on RNA alternative splicing. (a) Flowchart of alternative splicing analysis based on RNA-seq data (see details in Methods). (b) PSI values for Fig. 4f is displayed graphically. PSI values were measured three times by image J. (c) HEK293 cells as described in Fig. 4f were subjected to alternative splicing analysis for genes as indicated. The position of the cassette exon in each gene was as following: *ECSIT* (NM_001243204, exon6); *CCDC180* (NM_020893, exon19); *COLEC12* (NM_13038d6, exon2); *ZNF44* (NM_001353550, exon2); *MED12L* (NM_053002, exon11). (d) PSI values for (c) is displayed graphically. PSI values were measured three times by image J. (e) HEK293 cells as described in Fig. 4f were subjected to immunoblotting analysis to examine protein levels of PRMT4, 5, and 7. (f) HEK293 cells transfected with *siCTL* or *siPRMT4* and *siPRMT5* in the presence or absence of *siPRMT7* followed by alternative splicing analysis as described in Fig. 4f. (g) PSI values for (f) is displayed graphically. PSI values were measured three times by image J. (h) HEK293 cells as described in (f) were subjected to immunoblotting analysis to examine protein levels of PRMT4, 5, and 7. (i-k) PSI values for Fig. 4G (i), 4H (j), and 4I (k) is displayed graphically. PSI values were measured three times by image J. (l) HEK293 cells transfected with *siCTL* or *siPRMT1* were subjected to alternative splicing analysis. (m) PSI values for (l) is displayed graphically. PSI values were measured three times by image J. (n) HEK293 cells as described in Fig. 4g, 4h, 4i, and Supplementary Figure 4l were subjected to immunoblotting analysis to examine the protein levels of PRMT4, 5,7, and 1. Source data are provided as a Source Data file.

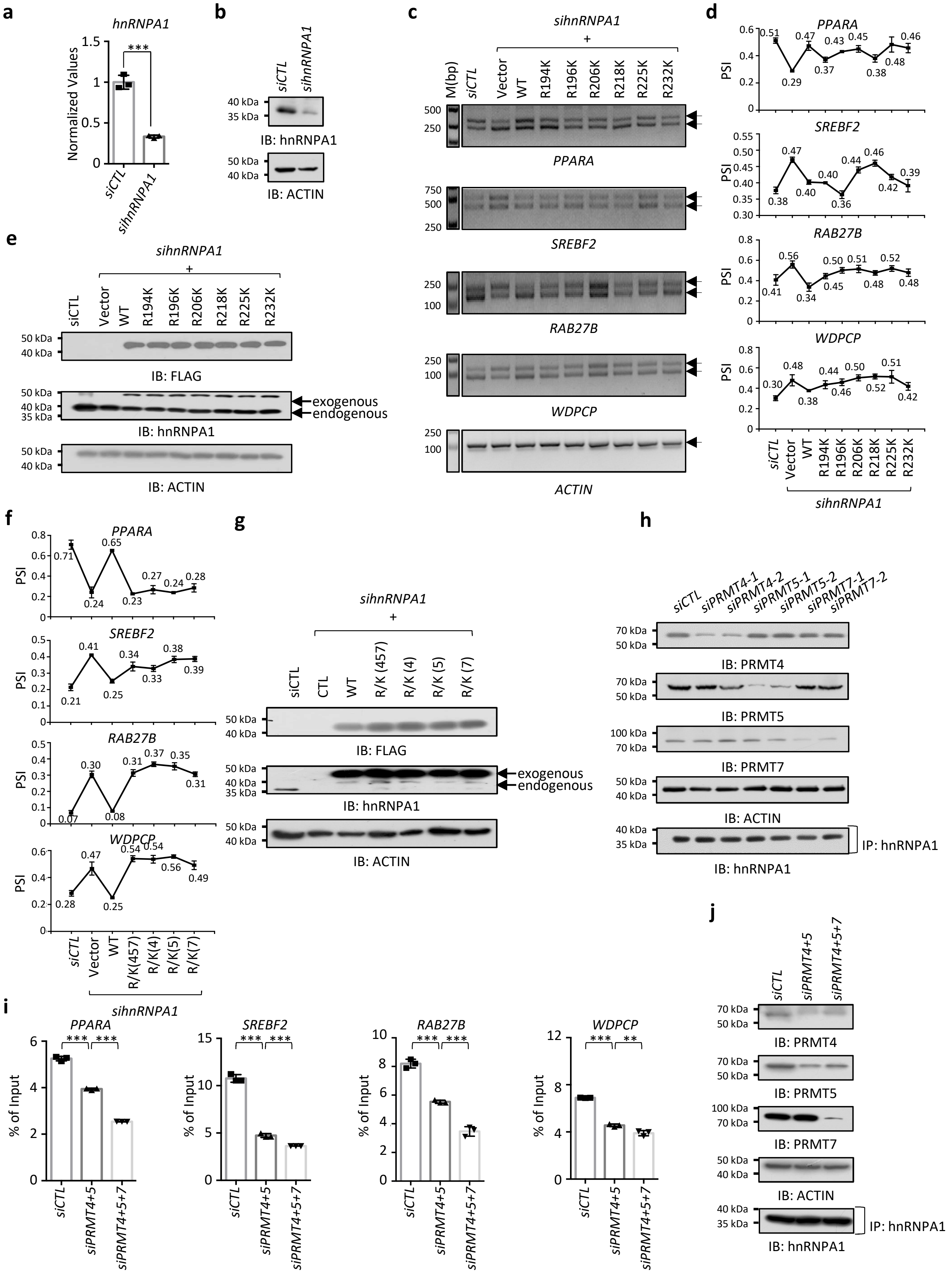

a
hnRNPA1
Normalized Values
***
siCTL
sihnRNPA1
b
siCTL
sihnRNPA1
40 kDa
35 kDa
IB: hnRNPA1
50 kDa
40 kDa
IB: ACTIN
c
sihnRNPA1
+
M(bp)
siCTL
Vector
WT
R194K
R196K
R206K
R218K
R225K
R232K
500
250
PPARA
750
500
250
SREBF2
250
100
RAB27B
250
100
WDPCP
250
100
ACTIN
d
PPARA
PSI
0.51
0.29
0.47
0.37
0.43
0.45
0.38
0.48
0.46
SREBF2
0.38
0.47
0.40
0.40
0.36
0.44
0.46
0.42
0.39
RAB27B
0.41
0.56
0.34
0.45
0.50
0.51
0.48
0.52
0.48
WDPCP
0.30
0.48
0.38
0.44
0.46
0.50
0.52
0.51
0.42
siCTL
Vector
WT
R194K
R196K
R206K
R218K
R225K
R232K
sihnRNPA1
e
sihnRNPA1
+
siCTL
Vector
WT
R194K
R196K
R206K
R218K
R225K
R232K
50 kDa
40 kDa
IB: FLAG
50 kDa
40 kDa
35 kDa
exogenous
endogenous
IB: hnRNPA1
50 kDa
40 kDa
IB: ACTIN
f
PPARA
PSI
0.71
0.24
0.65
0.23
0.27
0.24
0.28
SREBF2
0.21
0.41
0.25
0.34
0.33
0.38
0.39
RAB27B
0.07
0.30
0.08
0.31
0.37
0.35
0.31
WDPCP
0.28
0.47
0.25
0.54
0.54
0.56
0.49
siCTL
Vector
WT
R/K(457)
R/K(4)
R/K(5)
R/K(7)
sihnRNPA1
g
sihnRNPA1
+
siCTL
CTL
WT
R/K (457)
R/K (4)
R/K (5)
R/K (7)
50 kDa
40 kDa
IB: FLAG
50 kDa
40 kDa
35 kDa
exogenous
endogenous
IB: hnRNPA1
50 kDa
40 kDa
IB: ACTIN
h
siCTL
siPRMT4-1
siPRMT4-2
siPRMT5-1
siPRMT5-2
siPRMT7-1
siPRMT7-2
70 kDa
50 kDa
IB: PRMT4
70 kDa
50 kDa
IB: PRMT5
100 kDa
70 kDa
IB: PRMT7
50 kDa
40 kDa
IB: ACTIN
40 kDa
35 kDa
IP: hnRNPA1
IB: hnRNPA1
i
PPARA
SREBF2
RAB27B
WDPCP
% of Input
***
**
siCTL
siPRMT4+5
siPRMT4+5+7
j
siCTL
siPRMT4+5
siPRMT4+5+7
70 kDa
50 kDa
IB: PRMT4
70 kDa
50 kDa
IB: PRMT5
100 kDa
70 kDa
IB: PRMT7
50 kDa
40 kDa
IB: ACTIN
40 kDa
35 kDa
IP: hnRNPA1
IB: hnRNPA1

k

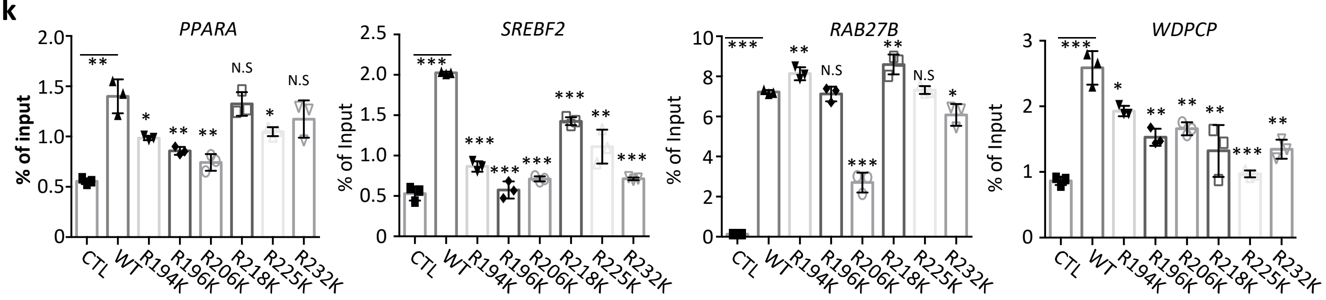
PPARA
SREBF2
RAB27B
WDPCP
% of input
% of Input
CTL
WT
R194K
R196K
R206K
R218K
R225K
R232K
N.S
*
**
***

l

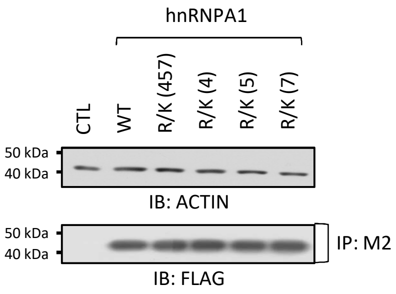
hnRNPA1
CTL
WT
R/K (457)
R/K (4)
R/K (5)
R/K (7)
50 kDa
40 kDa
IB: ACTIN
IP: M2
IB: FLAG

m

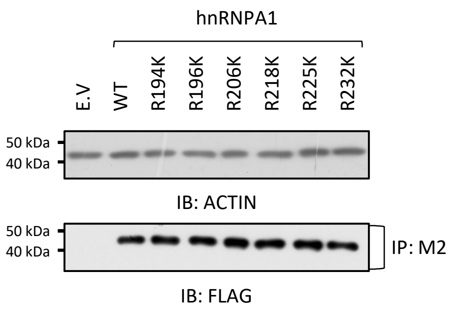
hnRNPA1
E.V
WT
R194K
R196K
R206K
R218K
R225K
R232K
50 kDa
40 kDa
IB: ACTIN
IP: M2
IB: FLAG

o

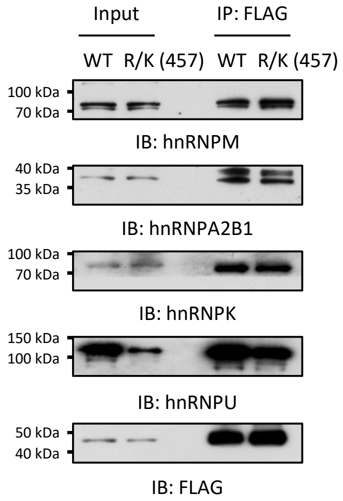
Input
IP: FLAG
WT
R/K (457)
100 kDa
70 kDa
IB: hnRNPM
40 kDa
35 kDa
IB: hnRNPA2B1
IB: hnRNPK
150 kDa
IB: hnRNPU
50 kDa
IB: FLAG

n

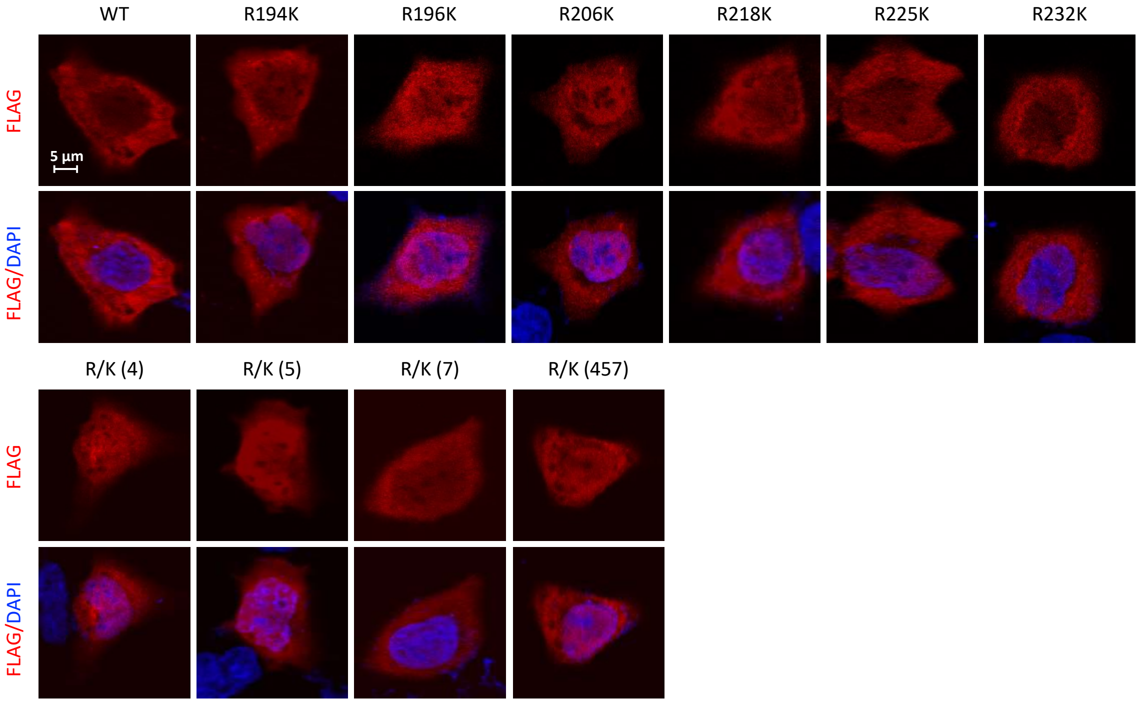
WT
R194K
R196K
R206K
R218K
R225K
R232K
FLAG
FLAG/DAPI
5 µm
R/K (4)
R/K (5)
R/K (7)
R/K (457)

Supplementary figure 5. PRMT4-, 5-, and 7-mediated hnRNPA1 methylation was involved in the regulation of alternative splicing events co-regulated by the three PRMTs. (a, b) HEK293 cells as described in Fig. 5d were subjected to RT-qPCR and immunoblotting. For RT-qPCR, n = 3 biological replicates (mean ± SEM, ***P < 0.001 by unpaired Student's t-test, two-tailed). (c) HEK293 cells transfected with *siCTL* or *sihnRNPA1* in the presence or absence of WT or mutants were subjected to alternative splicing analysis. (d) PSI values for (c) is displayed graphically. PSI values were measured three times by image J. (e) HEK293 cells as described in (c) were subjected to immunoblotting. (f) PSI values for Fig. 5f is displayed graphically. PSI values were measured three times by image J. (g) HEK293 cells as described in Fig. 5f were subjected to immunoblotting. (i) HEK293 cells transfected with *siCTL* or *siPRMT4* and *siPRMT5* in the presence or absence of *siPRMT7* followed by RNA-IP using IgG or anti-hnRNPA1 antibody. n = 3 biological replicates (mean ± SEM, *P < 0.05, **P < 0.01, ***P<0.001 by unpaired Student's t-test, two-tailed). (h, j) HEK293 cells as described in Fig. 5G (h) and (I) (j) were subjected to immunoblotting analysis. (k) HEK293 cells transfected with empty vector or Flag-tagged, WT or methylation mutants were subjected to RNA-IP using anti-Flag antibody. n = 3 biological replicates (mean ± SEM, *P < 0.05, **P < 0.01, ***P<0.001, N.S: non-significant, by unpaired Student's t-test, two-tailed). (l, m) HEK293 cells as described in Fig. 5h (l) and (k) (m) were subjected to immunoblotting. (n) HEK293 cells were transfected with hnRNPA1 (wt) or methylation mutants followed by immunofluorescence. (o) HEK293 transfected with Flag-tagged hnRNPA1 (wt) or R/K (457) mutant were subjected to immunoprecipitation followed by immunoblotting. Source data are provided as a Source Data file.

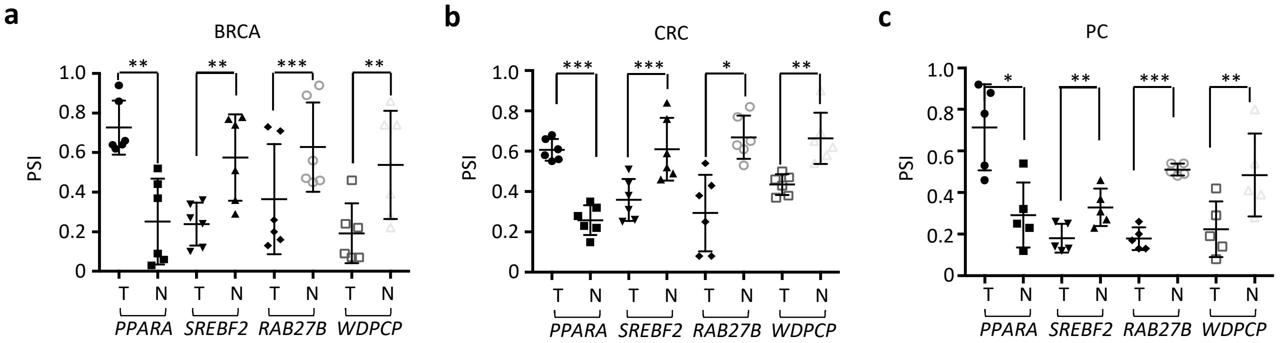

d

		PRMT4 (T/N)			*PRMT5* (T/N)			*PRMT7* (T/N)		
		BRCA	CRC	PC	BRCA	CRC	PC	BRCA	CRC	PC
PPARA (PSI (T/N))	BRCA	0.19	-	-	0.79	-	-	-0.06	-	-
	CRC	-	-0.43	-	-	-0.70	-	-	-0.53	-
	PC	-	-	0.04	-	-	-0.04	-	-	0.51
SREBF2 (PSI (T/N))	BRCA	-0.62	-	-	-0.28	-	-	-0.45	-	-
	CRC	-	0.50	-	-	-0.18	-	-	0.20	-
	PC	-	-	-0.30	-	-	-0.28	-	-	0.71
RAB27B (PSI (T/N))	BRCA	0.63	-	-	0.17	-	-	0.46	-	-
	CRC	-	-0.13	-	-	-0.47	-	-	-0.29	-
	PC	-	-	-0.27	-	-	-0.02	-	-	-0.16
WDPCP (PSI (T/N))	BRCA	0.15	-	-	-0.13	-	-	0.10	-	-
	CRC	-	-0.28	-	-	-0.45	-	-	-0.28	-
	PC	-	-	0.12	-	-	-0.06	-	-	-0.47

Supplementary figure 6. PRMT4, 5, 7, and hnRNPA1 arginine methylation were over-presented in multiple types of cancers. (a-c) PSI values for Fig. 6e-g is displayed graphically. n = 6 (e and g) or 5 (f) pairs of normal and tumor tissues (mean ± SEM, *P < 0.05, **P < 0.01, ***P < 0.001 by paired Student t-test, one-tailed). (d) Pearson correlation of the fold change of PRMT4, 5, and 7 expression between paired normal and tumor tissues with that of PSI. Source data are provided as a Source Data file.

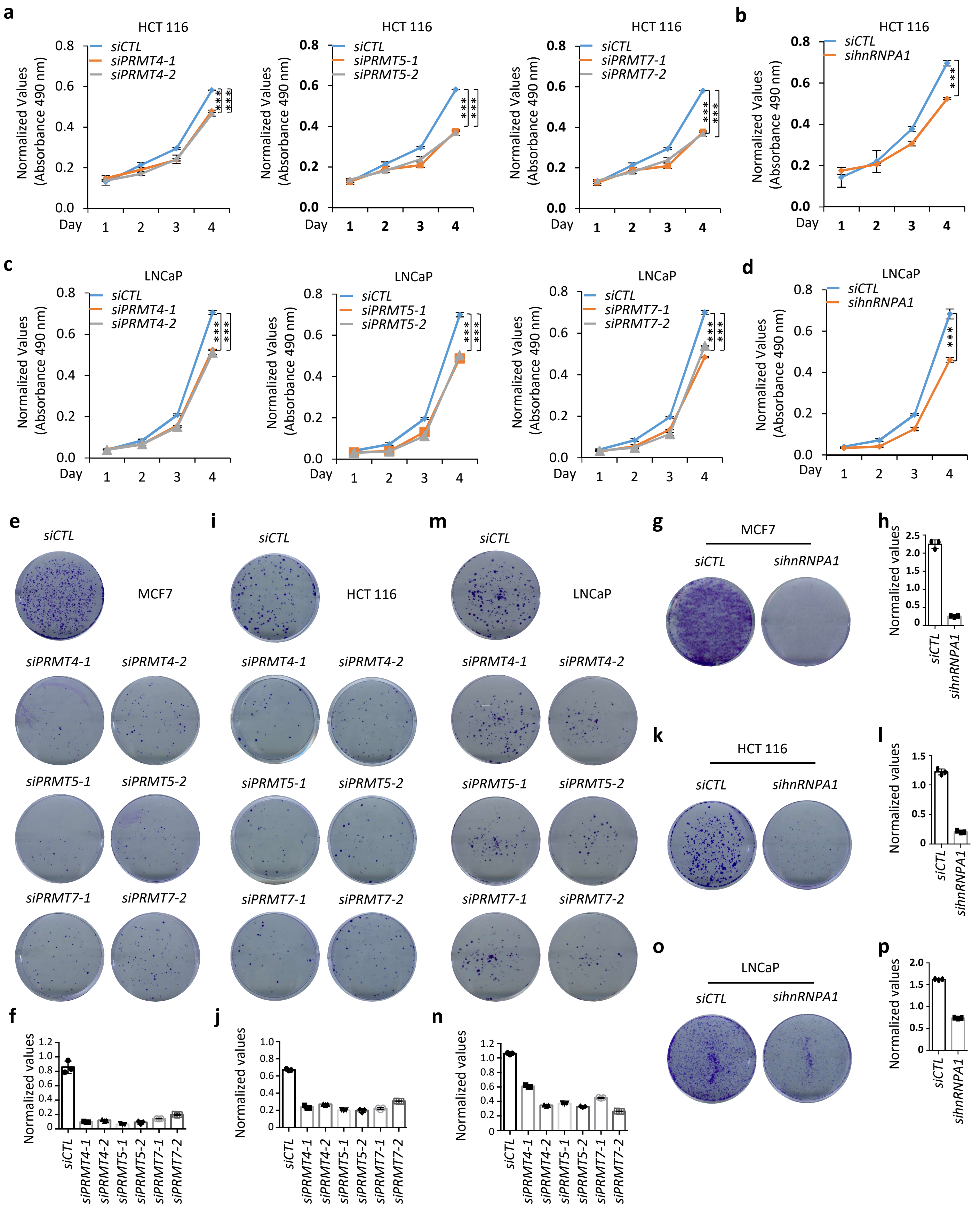
a
HCT 116
siCTL
siPRMT4-1
siPRMT4-2
siPRMT5-1
siPRMT5-2
siPRMT7-1
siPRMT7-2
Normalized Values
(Absorbance 490 nm)
Day
b
sihnRNPA1
c
LNCaP
d
e
MCF7
f
Normalized values
g
h
i
j
k
l
m
n
o
p
***

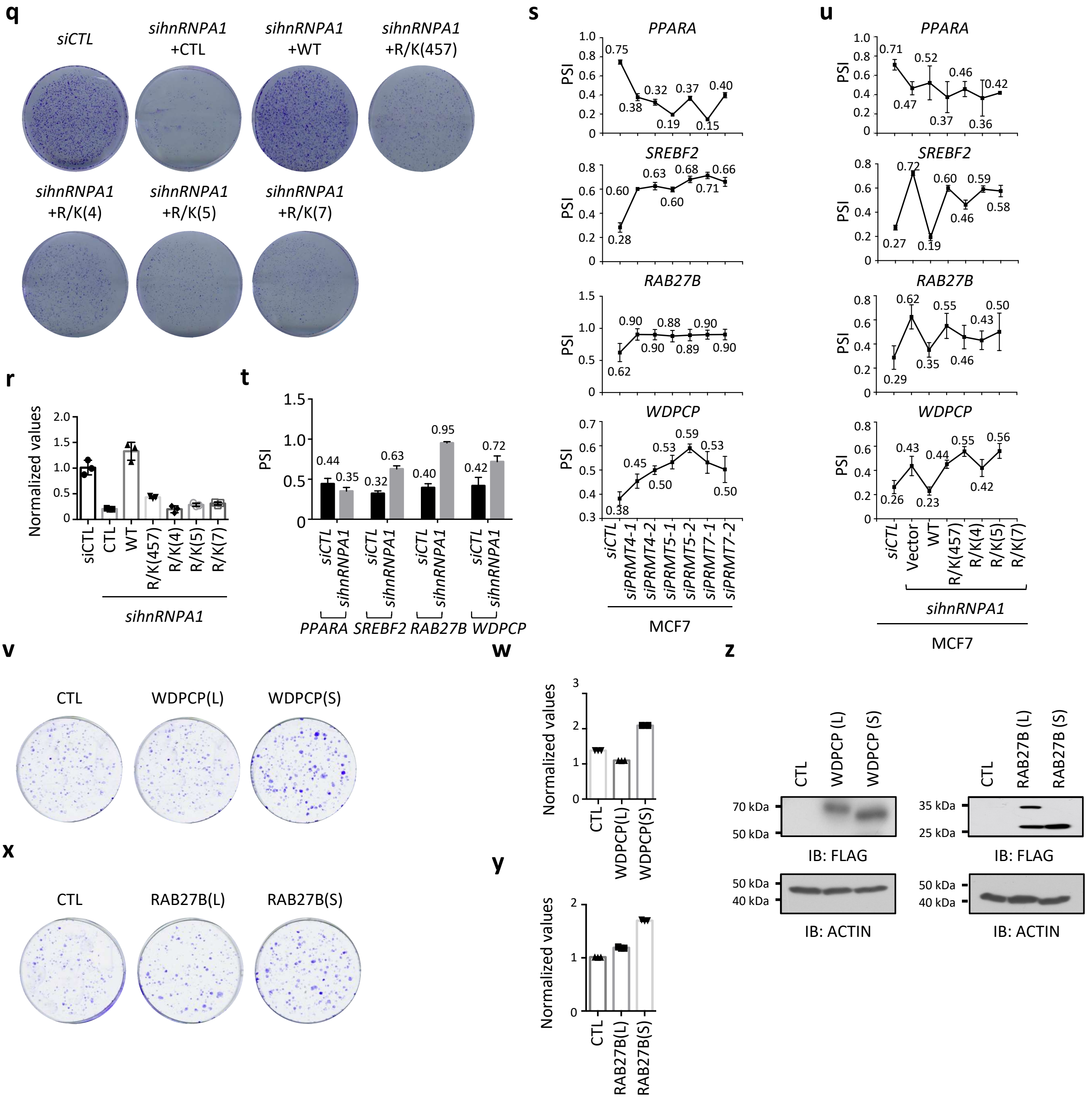
q
siCTL
sihnRNPA1 +CTL
sihnRNPA1 +WT
sihnRNPA1 +R/K(457)
sihnRNPA1 +R/K(4)
sihnRNPA1 +R/K(5)
sihnRNPA1 +R/K(7)
r
Normalized values
2.0
1.5
1.0
0.5
0
siCTL
CTL
WT
R/K(457)
R/K(4)
R/K(5)
R/K(7)
sihnRNPA1
t
PSI
1.5
1.0
0.5
0
0.44
0.35
0.32
0.63
0.40
0.95
0.42
0.72
siCTL
sihnRNPA1
PPARA
SREBF2
RAB27B
WDPCP
s
PPARA
PSI
0.75
0.38
0.32
0.19
0.37
0.15
0.40
SREBF2
0.60
0.28
0.63
0.60
0.68
0.71
0.66
RAB27B
0.62
0.90
0.90
0.88
0.89
0.90
0.90
WDPCP
0.38
0.45
0.50
0.53
0.59
0.53
0.50
siCTL
siPRMT4-1
siPRMT4-2
siPRMT5-1
siPRMT5-2
siPRMT7-1
siPRMT7-2
MCF7
u
PPARA
PSI
0.71
0.47
0.52
0.37
0.46
0.36
0.42
SREBF2
0.27
0.72
0.19
0.60
0.46
0.59
0.58
RAB27B
0.29
0.62
0.35
0.55
0.46
0.43
0.50
WDPCP
0.26
0.43
0.23
0.44
0.55
0.42
0.56
siCTL
Vector
WT
R/K(457)
R/K(4)
R/K(5)
R/K(7)
sihnRNPA1
MCF7
v
CTL
WDPCP(L)
WDPCP(S)
w
Normalized values
3
2
1
0
CTL
WDPCP(L)
WDPCP(S)
x
CTL
RAB27B(L)
RAB27B(S)
y
Normalized values
2
1
0
CTL
RAB27B(L)
RAB27B(S)
z
CTL
WDPCP (L)
WDPCP (S)
70 kDa
50 kDa
IB: FLAG
50 kDa
40 kDa
IB: ACTIN
CTL
RAB27B (L)
RAB27B (S)
35 kDa
25 kDa
IB: FLAG
50 kDa
40 kDa
IB: ACTIN

Supplementary figure 7. PRMT4, 5, 7, and hnRNPA1 arginine methylation were required for the growth of multiple types of cancer cells. (a, c, e, i, m) HCT-116 (a, i), LNCaP (c, m), or MCF7 cells were transfected with *siCTL* or *siPRMT4*, *5*, or *7* followed by cell proliferation assay (a, c) and colony formation assay (e, i, m). n = 3 biological replicates (mean ± SD, $^{**}P < 0.01$, $^{***}P < 0.001$ by unpaired Student's t-test, two-tailed). (b, d, g, k, o) HCT-116 (b, k), LNCaP (d, o), or MCF7 (g) cells were transfected with *siCTL* or *sihnRNPA1* followed by cell proliferation assay (b, d) and colony formation assay (g, k, o). n = 3 biological replicates (mean ± SD, $^{***}P < 0.001$ by unpaired Student's t-test, two-tailed). (f, h, j, l, n, p) Quantification of the crystal violet dye as shown in (e), (g), (i), (k), (m), and (o). Absorbance was measured three time. (q) MCF7 cells transfected with *siCTL* or *sihnRNPA1* in the presence or absence of wild type (WT) or methylation deficient mutants were subjected to colony formation assay. (r) Quantification of the crystal violet dye as shown in (q). Absorbance was measured three time. (s, t, u) PSI values for Fig. 7f, 7g and 7h is displayed graphically. PSI values were measured three times by image J. (v, x) MCF7 cells were transfected with empty vector (CTL) or vector expressing Flag-tagged WDPCP (w) or RAB27B (x), both short (S) and long (L) isoforms, followed by colony formation assay. (w, y) Quantification of the crystal violet dye as shown in (v) (w) and (x) (y). PSI values were measured three times by image J. (z) The expression of WDPCP (left) and RAB27B (right) isoforms was examined by immunoblotting. Source data are provided as a Source Data file.

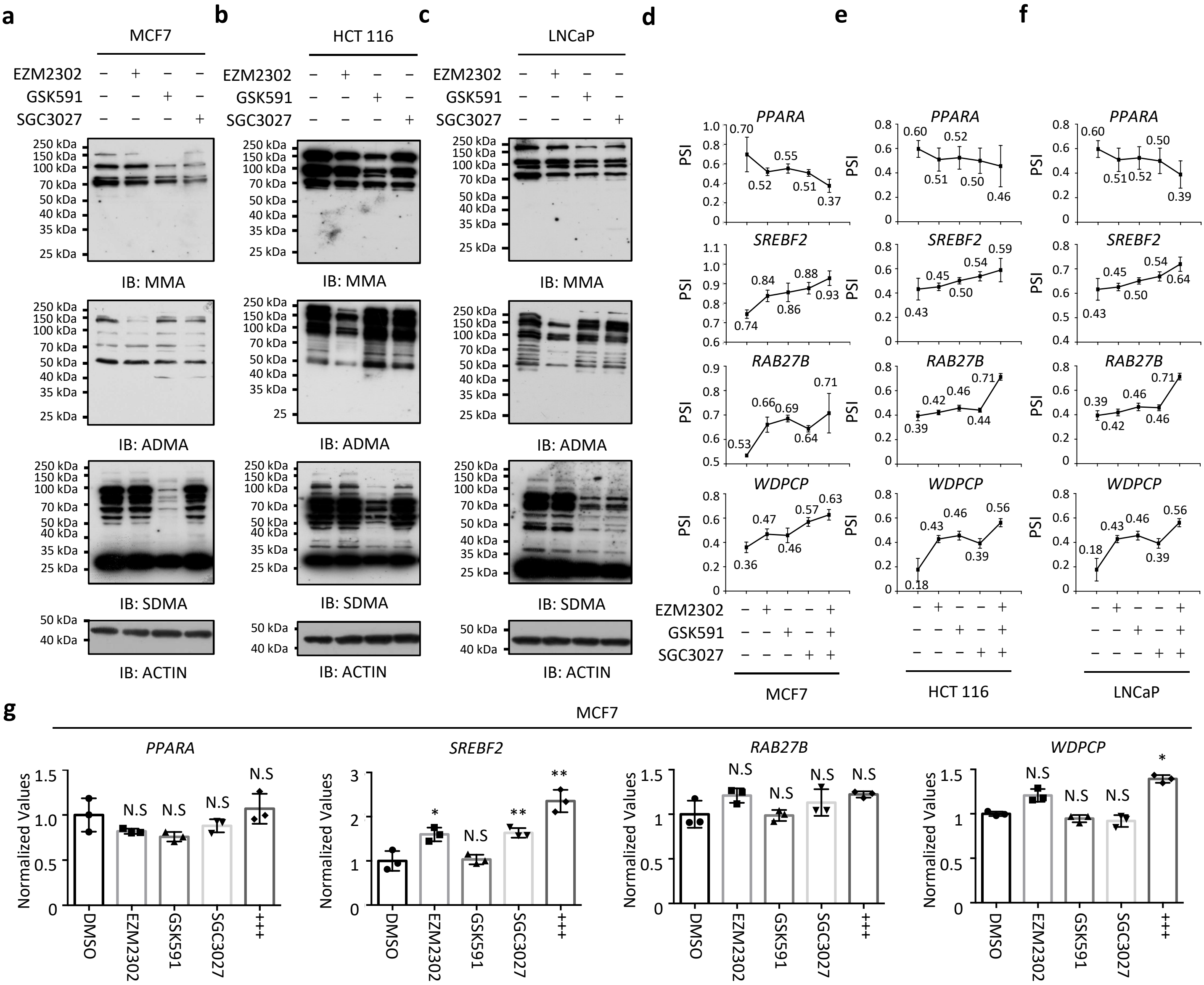

Supplementary figure 8. Pharmacological inhibition of PRMT4, 5, and 7 alters RNA alternative splicing and suppresses cancer cell growth. (a-c) MCF7 (a), HCT 116 (b), or LNCaP (c) cells were treated with EZM2302 (10 μM), GSK591 (10 μM), or SGC3027 (10 μM) alone or in combination for 48 hrs as indicated followed by immunoblotting. (d-f) PSI values for Fig. 8j-l is displayed graphically. PSI values were measured three times by image J. (g) MCF7 cells as described in Fig. 8j were subjected to RT-qPCR analysis to examine the expression of genes as indicated. n = 3 biological repeats (mean ± SEM, *P < 0.05, **P < 0.01, N.S: non-significant, by unpaired Student's t-test, two-tailed). Source data are provided as a Source Data file.
