# Supplementary material for: Profiling PRMT methylome reveals roles of hnRNPA1 arginine methylation in RNA splicing and cell growth
Source: Nat Commun. 2021 Mar 29;12:1946. doi: 10.1038/s41467-021-21963-1 (PMC8007824; doi:10.1038/s41467-021-21963-1)
Supplement: Supplementary file 2 — Description of Additional Supplementary Files [file 41467_2021_21963_MOESM2_ESM.docx]

File name: Supplementary Data 1.

Description: List of PRMT7-regulated arginine methylation sites. Raw output files from Proteome Discoverer (sheet 1 and 2), and proteins with arginine methylation sites on which mono-methylation signals decreased at least two-fold (sheet 3) or completely abolished (sheet 4) upon PRMT7 knockdown are shown.

File name: Supplementary Data 2.

Description: List of PRMT7-regulated proteins. Raw output files from Proteome Discoverer (sheet 1 and 2), and proteins of which abundance changed at least two-fold upon PRMT7 knockdown are shown (sheet 3).

File name: Supplementary Data 3.

Description: List of arginine methylation sites that were responsive to PRMT7 inhibitor SGC3027. Raw output file from Proteome Discoverer (sheet 1), and proteins with arginine methylation sites on which mono-methylation signals decreased at least two-fold (sheet 2) or completely abolished (sheet 3) upon SGC3027 incubation (10 μM, 48 hrs) are shown. PRMT7 substrates that are inhibited by SGC3027 are also shown (sheet 4).

File name: Supplementary Data 4.

Description: GO and KEGG pathway analysis for PRMT7 methylome. GO (sheet 1) and KEGG (sheet 2) pathway analysis for PRMT7 methylome were performed using Metascape^46^.

File name: Supplementary Data 5.

Description: List of PRMT4-regulated arginine methylation sites. Raw output file from Proteome Discoverer (sheet 1), and proteins with arginine methylation sites, mono- or asymmetric di-methylation, on which methylation signals decreased at least two-fold (sheet 2 and 4) or completely abolished (sheet 3 and 5) upon PRMT4 knockdown are shown.

File name: Supplementary Data 6.

Description: List of PRMT5-regulated arginine methylation sites. Raw output file from Proteome Discoverer (sheet 1), and proteins with arginine methylation sites, mono- or symmetric di-methylation, on which methylation signals decreased at least two-fold (sheet 2 and 4) or completely abolished (sheet 3 and 5) upon PRMT5 knockdown are shown.

File name: Supplementary Data 7.

Description: PRMT4-, PRMT5-, and PRMT7-commonly regulated methylated proteins. The 62 proteins that were commonly regulated by PRMT4, PRMT5, and PRMT7, and the methylation sites regulated in these proteins were listed.

File name: Supplementary Data 8.

Description: Arginine methylation sites in hnRNPA1 identified in clinical tissue samples. Four pairs of normal and tumor tissues from breast (BRCA), colon (CRC), and prostate (PC) were subjected to TMT (tandem mass tags) labeling and quantitative MS analysis followed by arginine methylation search. Raw output file for hnRNPA1 from Proteome Discoverer (sheet 1), and arginine methylation sites in the RGG domain of hnRNPA1 (sheet 2) are shown.

File name: Supplementary Data 9.

Description: Sequence information for all PCR primers used in the current study. Sequence information of qPCR primers designed to detect the gene expression or alternative splicing events were shown. F: forward; R: reverse.

File name: Supplementary Data 10.

Description: Comparison of PRMT7-interactome and substrates identified in different studies (*Mol Biol Cell* 30, 778-793; *Mol Biosyst* 9, 2231-2247; *Mol Cell Proteomics* 13, 2072-2088).

File name: Supplementary Data 11.

Description: Baseline characteristic for clinical samples included in this study.
